# The *Ncoa7* locus regulates V-ATPase formation and function, neurodevelopment and behaviour

**DOI:** 10.1007/s00018-020-03721-6

**Published:** 2020-12-19

**Authors:** Enrico Castroflorio, Joery den Hoed, Daria Svistunova, Mattéa J. Finelli, Alberto Cebrian-Serrano, Silvia Corrochano, Andrew R. Bassett, Benjamin Davies, Peter L. Oliver

**Affiliations:** 1 MRC Harwell Institute, Harwell Campus, Oxfordshire, OX11 0RD UK; 2 Department of Physiology, Anatomy and Genetics, University of Oxford, Parks Road, Oxford, OX1 3PT UK; 3 Wellcome Centre for Human Genetics, Roosevelt Drive, Oxford, OX3 7BN UK; 4 Present Address: Hospital Clínico San Carlos, Instituto de Investigación Sanitaria San Carlos, Calle del Prof Martín Lagos s/n, 28040 Madrid, Spain; 5 Wellcome Sanger Institute, Wellcome Genome Campus, Hinxton, Cambridge, CB10 1SA UK

**Keywords:** Lysosome, V-ATPase, Neuron, Mouse, Neurodevelopment, Behaviour, Autism

## Abstract

**Abstract:**

Members of the Tre2/Bub2/Cdc16 (TBC), lysin motif (LysM), domain catalytic (TLDc) protein family are associated with multiple neurodevelopmental disorders, although their exact roles in disease remain unclear. For example, nuclear receptor coactivator 7 (NCOA7) has been associated with autism, although almost nothing is known regarding the mode-of-action of this TLDc protein in the nervous system. Here we investigated the molecular function of NCOA7 in neurons and generated a novel mouse model to determine the consequences of deleting this locus in vivo. We show that NCOA7 interacts with the cytoplasmic domain of the vacuolar (V)-ATPase in the brain and demonstrate that this protein is required for normal assembly and activity of this critical proton pump. Neurons lacking *Ncoa7* exhibit altered development alongside defective lysosomal formation and function; accordingly, *Ncoa7* deletion animals exhibited abnormal neuronal patterning defects and a reduced expression of lysosomal markers. Furthermore, behavioural assessment revealed anxiety and social defects in mice lacking *Ncoa7*. In summary, we demonstrate that NCOA7 is an important V-ATPase regulatory protein in the brain, modulating lysosomal function, neuronal connectivity and behaviour; thus our study reveals a molecular mechanism controlling endolysosomal homeostasis that is essential for neurodevelopment.

**Graphic abstract:**

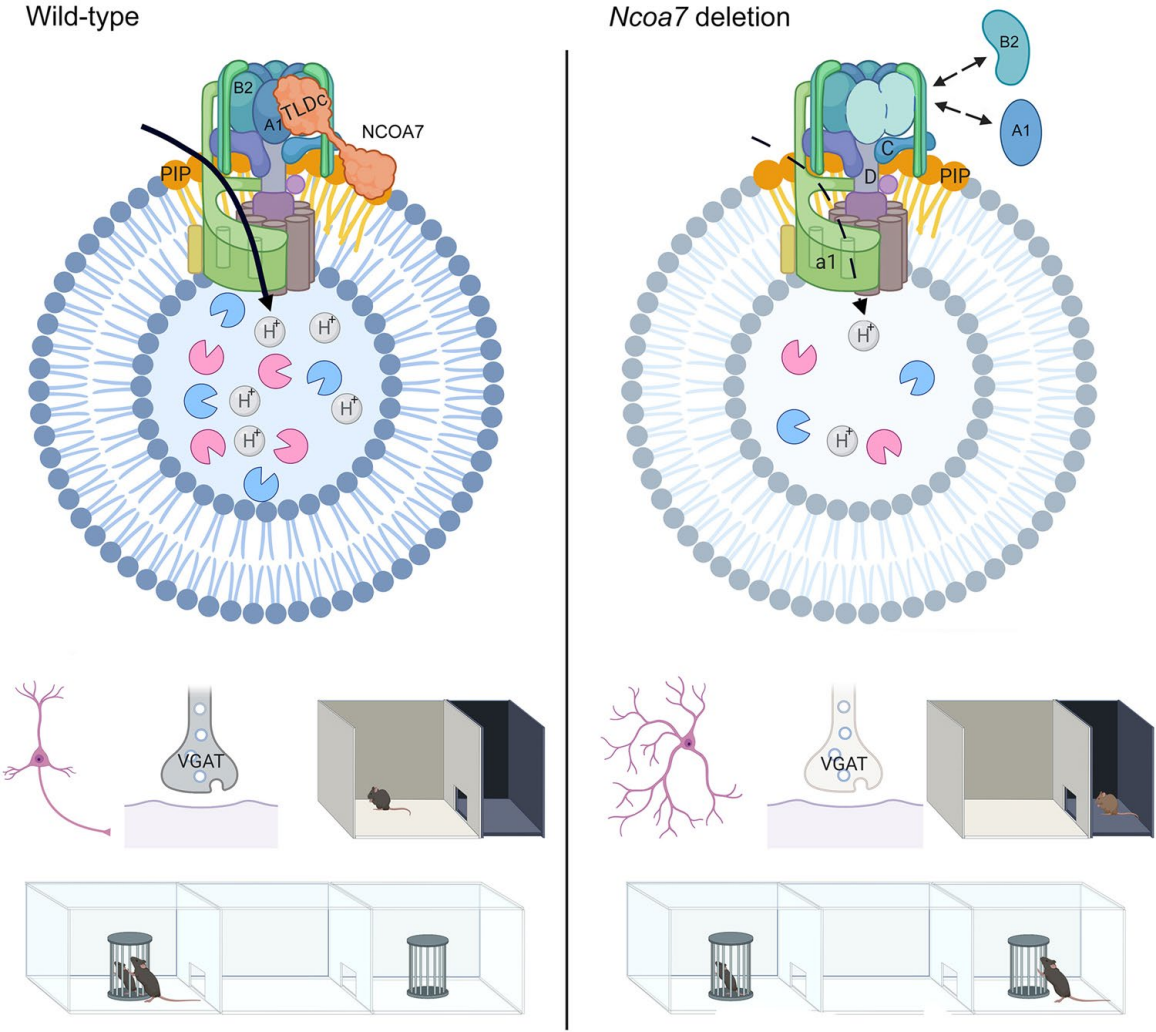

**Supplementary Information:**

The online version contains supplementary material available at 10.1007/s00018-020-03721-6.

## Introduction

The susceptibility to neurodevelopmental disorders (NDD) including autism spectrum disorder (ASD) is driven typically by multiple genetic components, and studies of rare cases have uncovered the importance of new mutations in the search for common molecular pathways and novel clinical interventions [1, 2]. Many of the candidate genes associated with NDD are clustered around known aspects of dendritic development and synapse formation [3, 4]; however, there is an increasing appreciation that fundamental, ubiquitous mechanisms of cellular maintenance are likely to play a key role. For example, neurons are highly dependent on autophagy and the endolysosomal system for their normal function and there are cases where the manipulation of such pathways can either be beneficial [5] or detrimental [6] to neurodevelopment and behaviour in mammalian systems. Specifically, neurons rely on efficient lysosomal degradative activity due to their postmitotic nature, cellular polarisation and structural complexity [7, 8]. Defective vesicular acidification, including the intraluminal pH of lysosomes, will ultimately influence the activity of degradative enzymes [9] and as such will be detrimental to the membrane and protein recycling events required for neuronal migration, dendritic elongation and neuronal connectivity, all aspects implicated in NDD [10–12].

One family of proteins increasingly associated with NDD share the highly-conserved Tre2/Bub2/Cdc16 (TBC), lysin motif (LysM), domain catalytic (TLDc) domain, initially implicated in the oxidative stress response [13, 14]. Since the advent of exome sequencing studies, causative mutations have been described in the TLDc proteins Tre2/Bub2/Cdc16 (TBC)1 domain family member 24 (TBC1D24) and oxidation resistance 1 (OXR1) in a range of NDD, some characterised by seizures and neurodegeneration as well as developmental delay [15–17]. Although both proteins have been proven to regulate aspects of neurotransmission, neurodevelopment and cellular survival in a number of model systems, their exact molecular function in disease remains unclear [13, 18–22]. A third member of the TLDc family highly-expressed in the central nervous system (CNS) is nuclear receptor coactivator 7 (NCOA7), and the most closely related to OXR1 [23]. NCOA7 was initially identified as estrogen receptor (ER)-associated protein 140 (ERAP140) and contains a putative ER binding domain [24, 25]; however, the role of this TLDc protein in genome-wide transcriptional regulation has not been demonstrated and almost nothing is known regarding the molecular function of the gene in the nervous system.

Interestingly, a large-scale ASD network study utilizing the BrainSpan human transcriptome database identified *NCOA7* as a ‘hub’ gene displaying one of the strongest interaction network correlations with disease [26]. Then, more recently, systematic analysis of exome data from the Autism Sequencing Consortium identified a recessive case of ASD caused by a homozygous nonsense mutation in *NCOA7* [27]. Thus, given the known associations of other TLDc genes with NDD and the lack of functional knowledge regarding NCOA7, here we generated a new mouse model to investigate the significance of *Ncoa7* loss-of-function for neurodevelopment and behaviour, and uncover the vital role of NCOA7 in endolysosomal homeostasis in neurons.

## Results

### Generation of a novel, viable *Ncoa7* deletion mouse model

To first establish the distribution of *Ncoa7* in the developing central nervous system, we carried out in situ hybridisation at a range of timepoints in wild-type (WT) mice. These data show that *Ncoa7* is widely expressed in all major regions of the brain, from embryonic and neonatal stages and into adulthood (Supplementary Figure S1). *NCOA7* is also expressed as a number of isoforms in mammalian tissues; several full-length containing all of the annotated domains (NCOA7), but also a much shorter isoform with a unique first exon followed by the TLDc domain alone (NCOA7-B; Supplementary Figure S2A) [13, 23, 28, 29]. Our previous work in the mouse has demonstrated that loss of all isoforms of the closely-related TLDc gene *Oxr1* is required to generate an overt neurological phenotype in vivo [30]. Therefore, to determine whether a similar phenomenon occurs for *Ncoa7*, and to investigate molecular mechanisms in the context of *Ncoa7* total loss-of-function, we generated a novel mouse knockout model in which the entire coding region was deleted by CRISPR-Cas9 (Supplementary Figure S2A). Mice homozygous for the approximate 157 kb deletion (*Ncoa7*^*del/del*^ or DEL, Supplementary Figure S2A-B) were viable and RT-PCR, in situ hybridisation and Western blotting confirmed that all *Ncoa7* isoforms are absent in DEL tissue (Supplementary Figure S2C–E). The gross brain structure was comparable between littermate WT and DEL animals, with no differences observed from quantitative morphometric analysis in adult mice (Supplementary Figure S2F–N). In addition, there was no indication of overt cell death in DEL brains as tested by caspase-3 staining up to 18 months of age (Supplementary Figure S2O).

### NCOA7 interacts with the V-ATPase in vivo and modulates complex assembly

The vacuolar (V)-ATPase is a multi-subunit ATP-dependent proton pump, comprising of cytosolic (V1) and integral membrane (V0) domains, that is vital for maintaining the acidic intraluminal pH of multiple cellular organelles [31]. In a screen for protein interactors of this complex in the kidney, NCOA7 was identified as a potential binding partner [32, 33]. In the nervous system, the V-ATPase plays a vital role in neuronal and synaptic physiology, with mutations in specific subunits causing neurodevelopmental disease [34, 35]. Therefore, in view of the recent association of *NCOA7* loss-of-function mutations in autism [27], and the distinct lack of knowledge regarding NCOA7 function in the nervous system, we investigated neuronal V-ATPase function in vivo using our new *Ncoa7* deletion model.

**Fig. 1:**
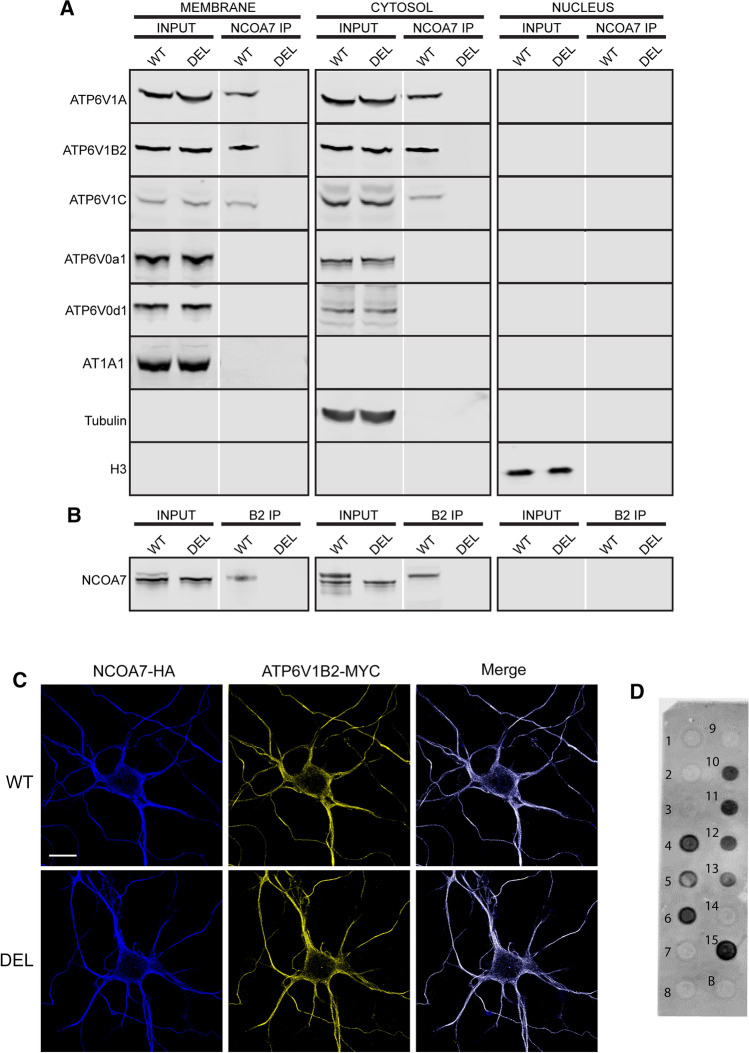
NCOA7 interacts specifically with V1 domain subunits of the V-ATPase. **a** Western blotting for V-ATPase V1 and V0 subunits after immunoprecipition (IP) of membrane, cytosolic and nuclear fractions from WT and DEL adult mouse whole brain using anti-NCOA7. Blots of equally loaded protein extracts prior to IP (input) are also shown. The markers AT1A1, tubulin and Histone H3 are used as loading controls for the membrane, cytosolic and nuclear fractions, respectively. **b** Western blot detection of NCOA7 after IP from the same adult mouse whole brain fractions using anti-ATP6V1B2. **c** Immunofluorescence for HA-tagged NCOA7 and myc-tagged ATP6V1B2 expressed in primary neurons (7–10 DIV) with co-localisation demonstrated by the merged signal. Scale bar: 20 µm. **d** PIP membrane showing the immunoreactive spots relative to positive NCOA7-PIP binding: *1* Lysophosphatidic acid; *2* Lysophosphatidylcholine; *3* Phospatidylinositol (PtdIns); *4* PtdIns(3)P; *5* PtdIns(4)P; *6* PtdIns(5)P; *7* Phosphatidylethanolamine; *8* Phosphatidylcholine; *9* Sphingosine 1-phosphate; *10* PtdIns(3,4)P2; *11* PtdIns(3,5)P2; *12* PtdIns(4,5)P2; *13* PtdIns(3,4,5)P3; *14* Phosphatidic acid; *15* Phosphatidylserine; *B* Blank

First, we assessed the endogenous protein–protein interactions of NCOA7 with individual subunits of the V-ATPase in vivo. Co-immunoprecipitation experiments from sub-fractionated WT brain tissue using an anti-NCOA7 antibody demonstrated an interaction of the protein with the cytosolic V1 subunits ATP6V1B2, ATP6V1A(1) and ATP6V1C, with DEL mice samples used to confirm the specificity of the binding (Fig. 1a). In addition, we confirmed the NCOA7:ATP6V1B2 interaction by reciprocal co-immunoprecipitation with an anti-ATP6V1B2 antibody in both the cytoplasmic and membrane fractions (Fig. 1b). Interestingly, however, an interaction with the integral membranous V0 subunits ATP6V0a1 and ATP6v0d1 could not be detected (Fig. 1a). Before investigating the functional consequences of this interaction in the context of *Ncoa7* disruption, we next measured the expression levels of a range of V1 and V0 V-ATPase subunits in whole brain tissue from DEL mice, and no significant differences were found at the protein level (Supplementary Figure S3A–F).

NCOA7 has been described as being nuclear-localised [23, 24], yet the Rab-like GTPase activators and myotubularins (GRAM) domain, present in the N-terminal region, is found in a number of membrane-associated proteins [36]. Therefore, to further support the interaction of NCOA7 with a membrane-associated protein complex, and in view of the lack of reliable antibodies for immunocytochemistry, we co-expressed NCOA7 and ATP6V1B2 in primary neurons. Immunostaining showed a positive signal for NCOA7 in the cytoplasm and at the membrane in both WT and DEL cells that co-localised with the ATP6V1B2 subunit (Fig. 1c). Indeed, our data showing a lack of detectable endogenous NCOA7 in the nuclear fraction of brain tissue, but robust expression in the cytoplasmic and membrane fractions, supports these data (Fig. 1a). In addition, we investigated whether NCOA7 is able to interact with membrane-associated phosphoinositides (PIPs), given their known importance for V-ATPase functionality and known physical proximity to the complex [37–39]. Using a protein-lipid overlay assay, we discovered that recombinant NCOA7 can bind specifically to a range of PIPs (Fig. 1d); importantly, those found to interact are known to be required for correct localisation and activity of the V-ATPase [37–39].

**Fig. 2:**
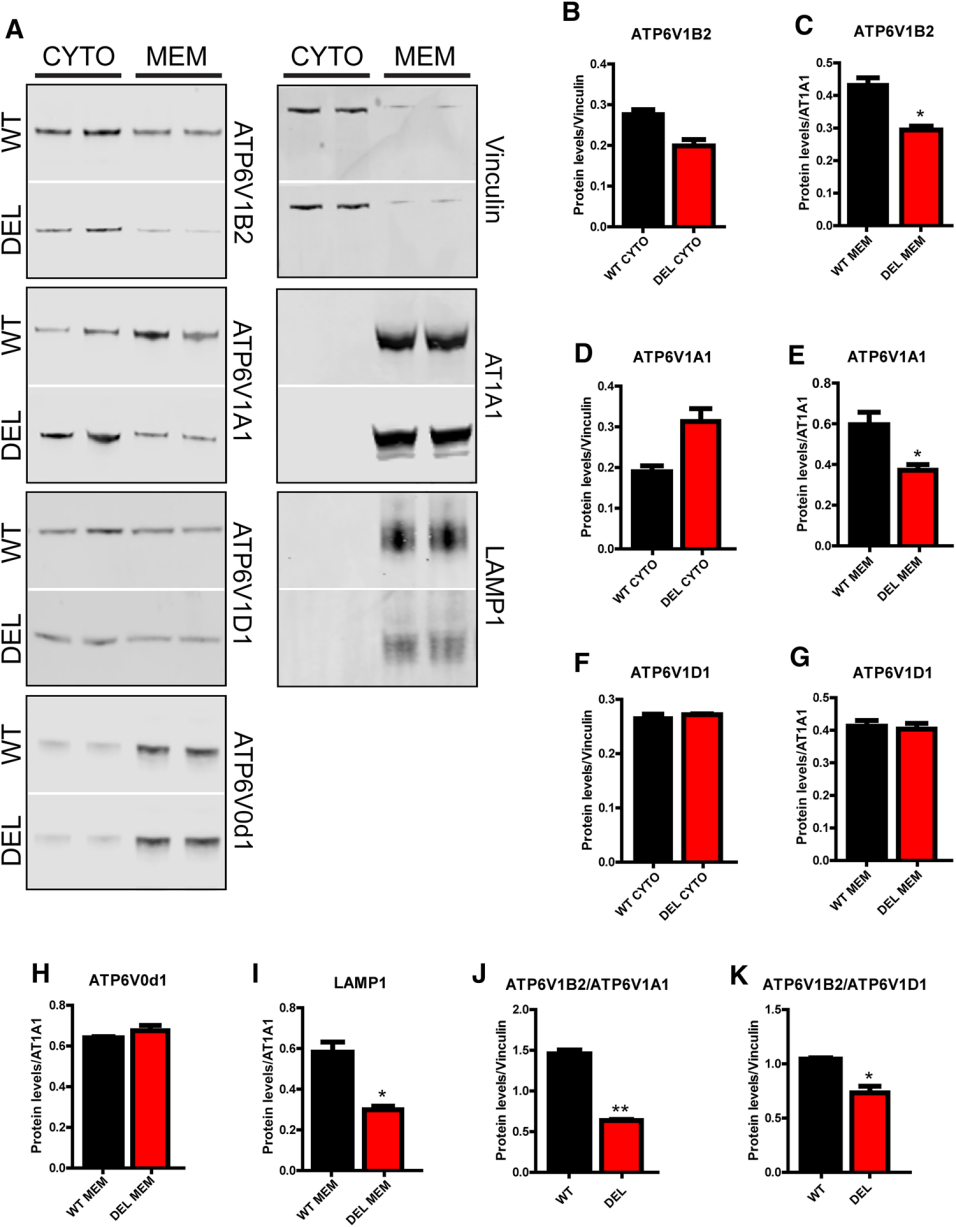
NCOA7 modulates V-ATPase V1/V0 complex assembly. **a** Primary cortical cell homogenates from WT and DEL mice were separated into cytosolic (CYTO) and membrane (MEM) fraction by sedimentation and subjected to Western blotting using antibodies against the V1 or V0 subunits as shown. The markers vinculin and AT1A1 are used as loading controls for the cytosolic and membrane fractions, respectively. **b** Quantification of cytosolic ATP6V1B2 versus vinculin and **c** membrane ATP6V1B2 versus AT1A1 from both genotypes. **d** Total quantification of cytosolic ATP6V1A1 versus vinculin and **e** membrane ATP6V1A1 versus AT1A1 from both genotypes. **f** Total quantification of cytosolic ATP6V1D1 versus vinculin and **g** membrane ATP6V1A1 versus AT1A1 from both genotypes. **h**, **i** Total quantification of ATP6V0d1 and LAMP1 versus the membrane loading control AT1A1. **j**, **k** Expression ratios of ATP6VA1 and ATP6V1D1 versus ATP6V1B2. Data are expressed as the the mean ± SEM from *n* = 4 independent cell preparations. *p < 0.05, **p < 0.01; unpaired Student’s *t *test

The correct assembly of the V-ATPase is essential for normal function of the complex, thus the reversible association and dissociation of the V1 and V0 subunits is an important regulatory feature [39, 40]. Given the apparent specificity of NCOA7 for the cytoplasmic subunits of the V-ATPase, we decided to study whether loss of *Ncoa7* would affect the assembly of the V1 domain on the membranous V0 domain. Subcellular fractionation experiments from WT and DEL primary cortical neurons did not reveal any differences in the protein levels of the V-ATPase subunits in the cytosolic fraction, consistent with our data from brain tissue (Fig. 2a, b, d, f). Interestingly, we found a significant reduction in the amount of the V1 subunits ATP6V1B2 and ATP6V1A1 present in the membrane fraction of DEL neurons compared to WT (Fig. 2c, e), although no such reduction was found for ATP6V1D (Fig. 2g). Furthermore, the level of the V0 subunit ATP6V0d1 was also unaltered between genotypes in the membrane fraction (Fig. 2h). Together, these findings reveal that NCOA7 is an interactor of the V1 domain of the V-ATPase in the brain, such that, in the absence of all *Ncoa7* isoforms, the ATP6V1B2 and ATP6V1A1 subunits may not be correctly stabilised at membrane sites where fully functional V-ATPases are maintained.

### *Ncoa7* deletion results in lysosomal dysfunction in neurons

**Fig. 3:**
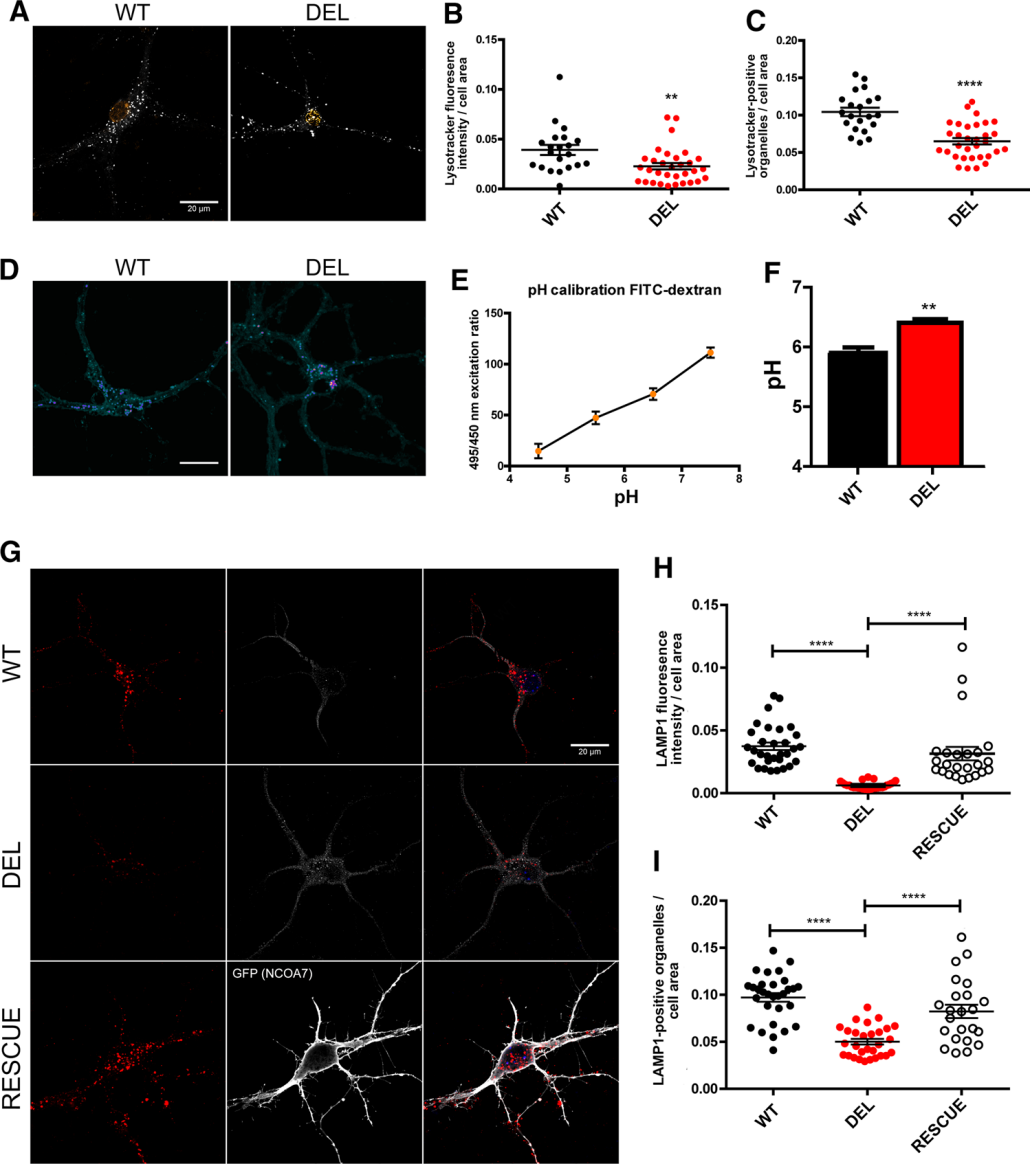
Ncoa7 deletion increases lysosomal pH and reduces lysosomal density in cortical neurons. **a** Representative images of LysoTracker Red live-labeled acidic organelles in WT and DEL primary cortical neurons (10–12 DIV) with DAPI. Scale bar: 20 µm. **b** LysoTracker fluorescence intensity and **c** LysoTracker-positive organelles quantification from WT and DEL primary cortical neurons. **d** Representative images of FITC-dextan live-labelled primary cortical neurons (7–10 DIV) from WT and DEL mice. Scale bar: 20 µm. **e** Calibration curve using pH standards used to calculate acidity of FITC-dextran live-labelled organelles (total *n* = 167). **f** pH quantification of WT (total *n* = 122) and DEL (total *n* = 140) live-labelled oranelles using the standard curve. **g** Representative images of LAMP1 staining (red, left panels), plasma membrane staining (middle panels) and their merged signals (right panels) in WT, DEL, and the RESCUE experiment from DEL neurons expressing exogenous NCOA7-GFP (lower panels). Scale bar: 20 µm. **h** LAMP1 fluorescence intensity and **i** the number of LAMP1-positive organelles was also quantified. Data are expressed as the mean ± SEM. Data points represent individual cells and *n* = 3 independent primary cell preparations were used for all panels. *p < 0.05, **p < 0.01, ***p < 0.001, ****p < 0.0001; unpaired Student’s *t *test (**b**, **c**, **f**) and one-way ANOVA/Bonferroni’s multiple comparison test (**h**, **i**)

Lysosomal V-ATPases are critical for regulating the optimal interluminal pH for enzymes, such as cathepsins and hydrolases, to facilitate the degradation of macromolecules derived from endocytic and autophagic pathways [41]. Thus to determine whether the loss of the NCOA7 interaction with the V-ATPase is critical for normal lysosomal function in neurons, we labelled the endolysosomal pathway using the pH-sensitive LysoTracker dye [41]. Live labelling in primary cortical cells revealed a reduction in the number of LysoTracker positive organelles in DEL cells compared to WT, accompanied by a significant decrease of their fluorescence signal (Fig. 3a–c). These data are suggestive of a change in lysosomal function; but to more accurately quantify the acidity of endocytic compartments, we loaded primary cells with pH-responsive FITC-dextran followed by live cell imaging, revealing a significant increase in pH of over 0.5 units in DEL neurons compared to WT controls (Fig. 3d–f).

**Fig. 4:**
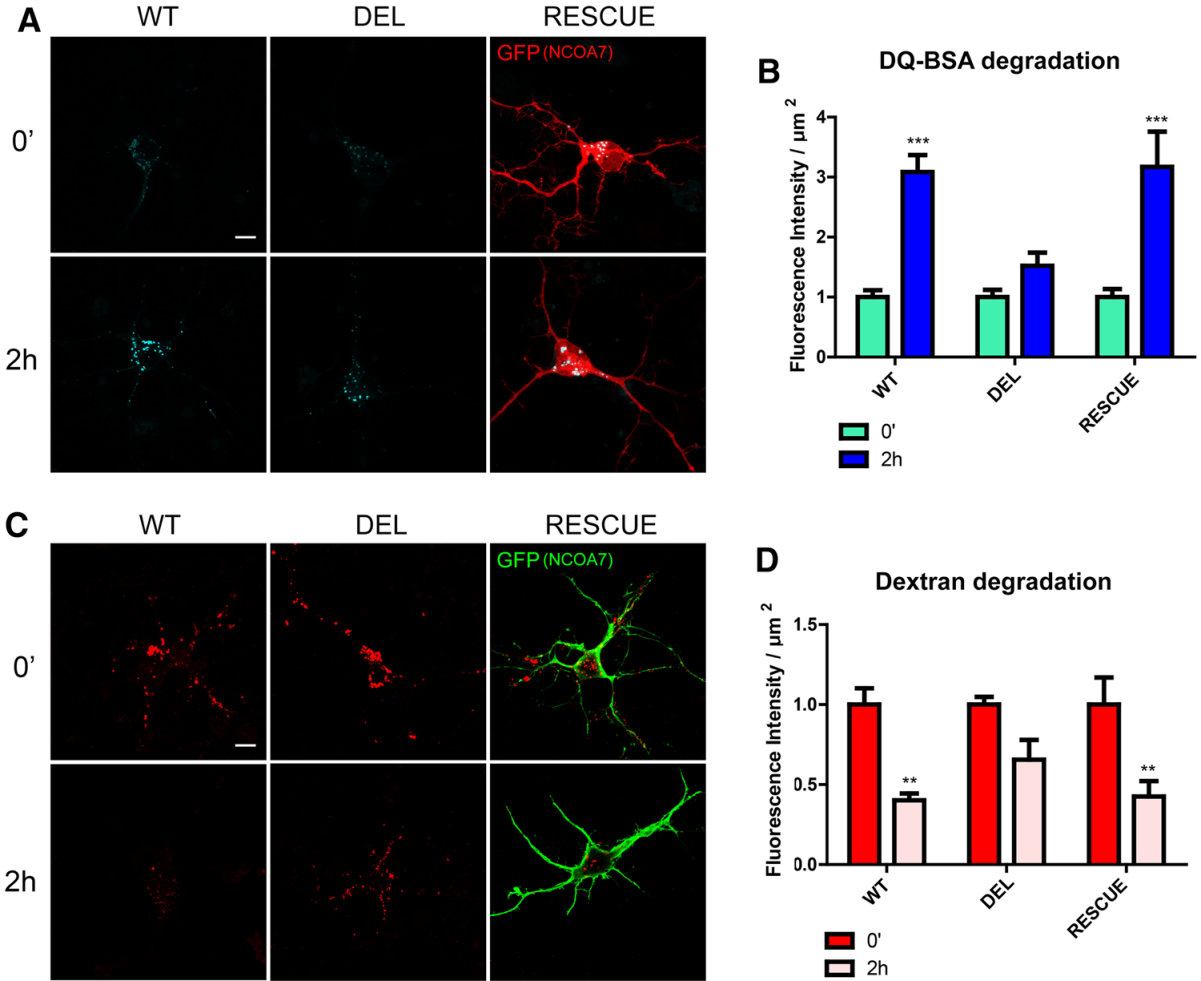
*Ncoa7* deletion reduces lysosome degradative activity. **a** Representative WT and DEL primary cortical neurons (10–12 DIV) and the relative RESCUE experiment (DEL neurons expressing exogenous full-length NCOA7) loaded with the fluorogenic substrate for lysosomal proteases DQ-Red-BSA and live-imaged at the time points indicated. Scale bar: 20 µm. **b** DQ-BSA de-quenching was quantified at different time points to analyse hydrolisation. **c** Representative WT, DEL and RESCUE neurons loaded with Dextran-AlexaFluor 594 and live-imaged at the time points indicated. Scale bar: 20 µm. **d** Dextran degradation was quantified by analysing quenching as a result of lysosomal degradative activity. Data are expressed as the mean ± SEM from *n* = 3 independent primary cell preparations. **p < 0.01, ***p < 0.001; one-way ANOVA/Bonferroni’s multiple comparison test

**Fig. 5:**
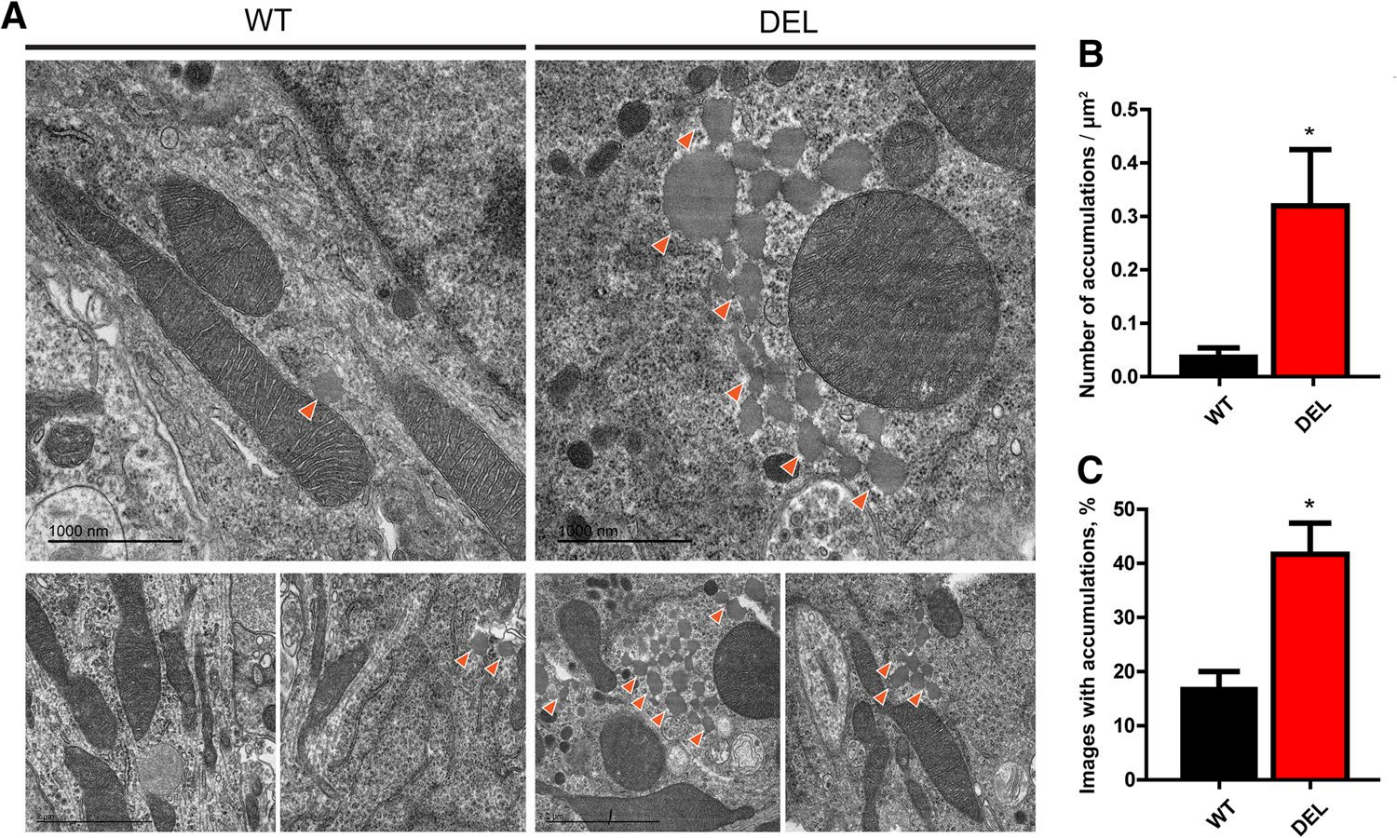
Ultrastructural analysis of undegraded material in WT and NCOA7-DEL primary cortical neurons. **a** Representative micrographs obtained from WT (left panel) and DEL (right panel) primary neurons. **b** Orange arrows highlight the granule-like structures that accumulate in the neuronal soma in DEL neurons. Scale bar: 1000 nm. **c**, **d** Quantification of the density (**c**) and percentage of accumulation-positive micrographs (**d**). Data are expressed as the mean ± SEM; *n* = 75 and *n* = 83 micrographs for WT and DEL neurons, respectively, from *n* = 3 independent preparations. *p < 0.05; one-way ANOVA/Bonferroni’s multiple comparison test

We next analysed LAMP1-positive organelles as a quantifiable marker of lysosomes by immunocytochemistry. A significant reduction in the number of LAMP1-positive puncta was detected in DEL primary neurons versus WT cells, in parallel with significantly lower LAMP1 fluorescence intensity in mutant cells (Fig. 3g–i). Crucially, overexpressing NCOA7 in DEL neurons was sufficient to rescue both phenotypes to WT values (Fig. 3h, i). Next, to further quantify lysosomal function in DEL cells, we utilised two independent assays in which the degradation of ectopically introduced compounds was measured [42, 43]. These data showed a significant reduction in the processing of both exogenous DQ-BSA and Dextran-AF594 in DEL primary cortical cells compared to WT, revealing reduced lysosomal degradative capacity in mutant neurons (Fig. 4a–d). Lysosomal defects are known to lead to cytosolic accumulation of undegraded material in post-mitotic neuronal cells [44, 45]. For this reason, we investigated whether the lysosomal alterations found in NCOA7 DEL mutant cells may lead to cytosolic accumulations in the neuronal soma. Conventional transmission electron microscopy on cell bodies of primary cortical neurons confirmed a signficant increase in the presence of cytosolic granules in DEL neurons versus WT, suggesting an accumulation of non-degraded material is due to the loss of *Ncoa7* (Fig. 5a–c). Taken together, these findings reveal that NCOA7 is required for normal lysosomal function in neurons.

Lysosomal function is intimately linked to autophagy, as lysosomes are targeted by autophagosomes to degrade engulfed materials. We, therefore, investigated the autophagy process by labelling LC3b and p62 in cultured primary cortical neurons. Neurons derived from DEL mice displayed a small but significant increase in the number of LC3b-positive organelles versus WT cells (Supplementary Figure S4A–C), while the number p62-positive were unaffected (Supplementary Figure S4D, E). Increased LC3b can be indicative of increased autophagosome formation or a decreased autophagic turnover, resulting in a build-up of LC3b-labelled autophagosomes. Given these observations in primary neurons, we questioned next whether the absence of *Ncoa7* may cause detectable lysosomal alterations in vivo. We examined the protein levels of endolysosomal markers from WT and DEL whole brain extracts. The endosomal marker Rab7, together with the lysosomal marker LAMP1 and the lysosomal enzymes cathepsin B and cathepsin D, were significantly decreased as observed by Western blot analysis (Supplementary Figure S5A–F). These data are further supported by an even more pronounced reduction in LAMP1 protein expression in the membrane fraction of primary neurons from DEL mice compared to WT controls (Fig. 2a, i). Overall, our findings reveal that loss of *Ncoa7* affects several aspects of endolysosomal physiology and homeostasis in neuronal cells, consistent with aberrant V-ATPase function or formation.

### V-ATPase-associated defects in the kidney from mice lacking Ncoa7

As stated above, an association between the V-ATPase and NCOA7 had been postulated from a protein interaction screen in the mouse kidney [32]. Thus we wanted to determine whether our data confirming NCOA7 binding and the influence on V-ATPase-associated function could be recapitulated in the kidney using our new DEL model. We began by showing that the kidney-specific ATP6V1B1 V1 subunit could successfully and specifically bind to NCOA7 in vivo by co-immunoprecipitation (Supplementary Figure S6A, B). Next, we assessed the expression of ATP6V1B1 in the kidney; interestingly, unlike V1 subunits in the brain, we were able to detect a significant reduction of protein levels in DEL tissue compared to WT (Supplementary Figure S6C). Importantly, the acidification of urine via the intercalated cells of the kidney relies on the correct functionality of the V-ATPase [46]. Therefore, we went-on to compare urine pH levels in WT and DEL mice. These data revealed a significant increase in urine pH from a large cohort of DEL animals (Supplementary Figure S6D); these findings are not only consistent with aberrant V-ATPase function in the kidney, but are also in agreement with the directionality of our lysosomal acidity measurements in primary neurons (Fig. 3F).

### Loss of *Ncoa7* impacts neuronal development and synaptic contacts in vivo

**Fig. 6:**
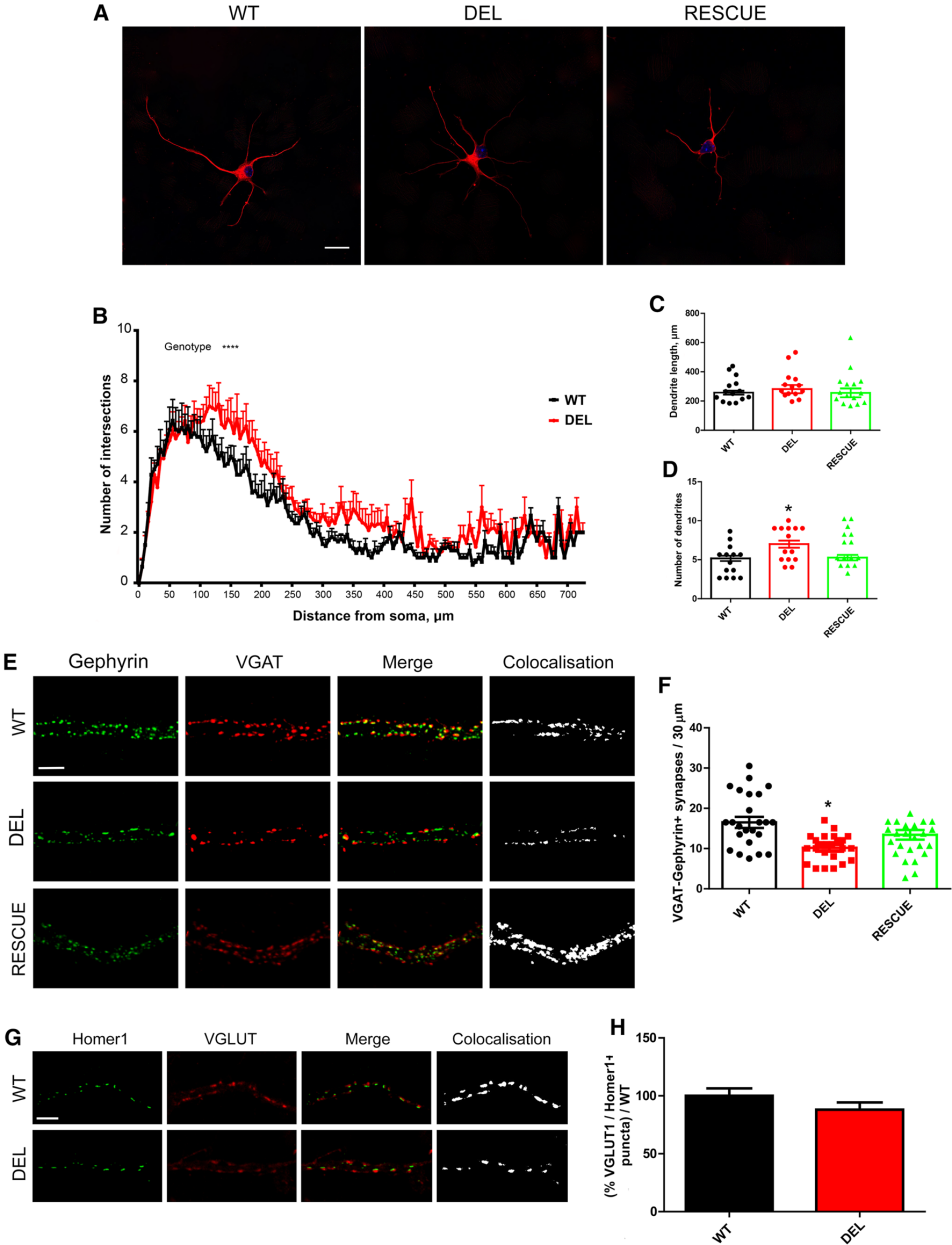
*Ncoa7* deletion alters neuronal development and decreases inhibitory synapse density in primary neruons. **a** Representative confocal microscopy images of WT and DEL primary cortical neurons (5-DIV) and the relative RESCUE experiment (DEL neurons expressing exogenous NCOA7) labelled with β3-tubulin. Scale bar: 20 μm. **b** Sholl analysis of cortical neuron arborisation as a function of distance from the soma. **c** Quantification of neurite length and **d** number from the Sholl analysis. **e** Representative high-magnification of proximal dendrites from cortical neurons. The black-and-white panels show the positive colocalisation between VGAT and Gephyrin along branches of WT, DEL and RESCUE neurons. Scale bar: 5 μm. **f** Quantitative analysis of synaptic punta counted on 30 μm branches starting from the cell body. **g** Representative images of proximal dendrites from primary cortical neurons. The black-and-white panels show the positive colocalisation between VGLUT1 and Homer1 along branches of WT and DEL neurons. Scale bar: 5 μm. **h** Quantitative analysis of VGLUT/Homer1-positive puncta counted on 30 μm branches starting from the cell body. Data are expressed as the mean ± SEM from *n* = 3 independent primary cell preparations. *p < 0.05; two-way ANOVA/Bonferroni’s multiple comparison test (**b**), one-way ANOVA/Bonferroni’s multiple comparison test (**c**, **d**) and unpaired Student’s *t *test (**f**–**h**)

Lysosomal degradative processes are essential for neuronal function and play key roles in nerve cell development and connectivity [47]. Therefore, as the role of NCOA7 in neurodevelopment has not been studied previously, we began by examining neurite extension and arborisation in DEL primary cortical cells. While the total length of the neuronal process were unaltered between genotypes (Fig. 6a–c), we found a significant increase in the number of proximal neurites in DEL neurons compared to WT, a phenotype that we were able to rescue by expressing exogenous NCOA7 (Fig. 6d). Later in their developmental stages, neurons establish functional networks balancing excitatory and inhibitory inputs. By measuring the juxtaposition of the presynaptic and postsynaptic inhibitory markers (VGAT and Gephyrin, respectively), we also uncovered a reduction of inhibitory contacts in DEL neurons when compared to WT (Fig. 6e, f). Importantly, this reduction could be rescued by the addition of exogenous NCOA7 (Fig. 6e, f); these data, therefore, revealing a fundamental role for NCOA7 in synaptic connectivity. On the contrary, when we analysed excitatory synapses by labelling with VGLUT1 and Homer1 on WT and DEL neurons, no statistical differences were found (Fig. 6g, h).

**Fig. 7:**
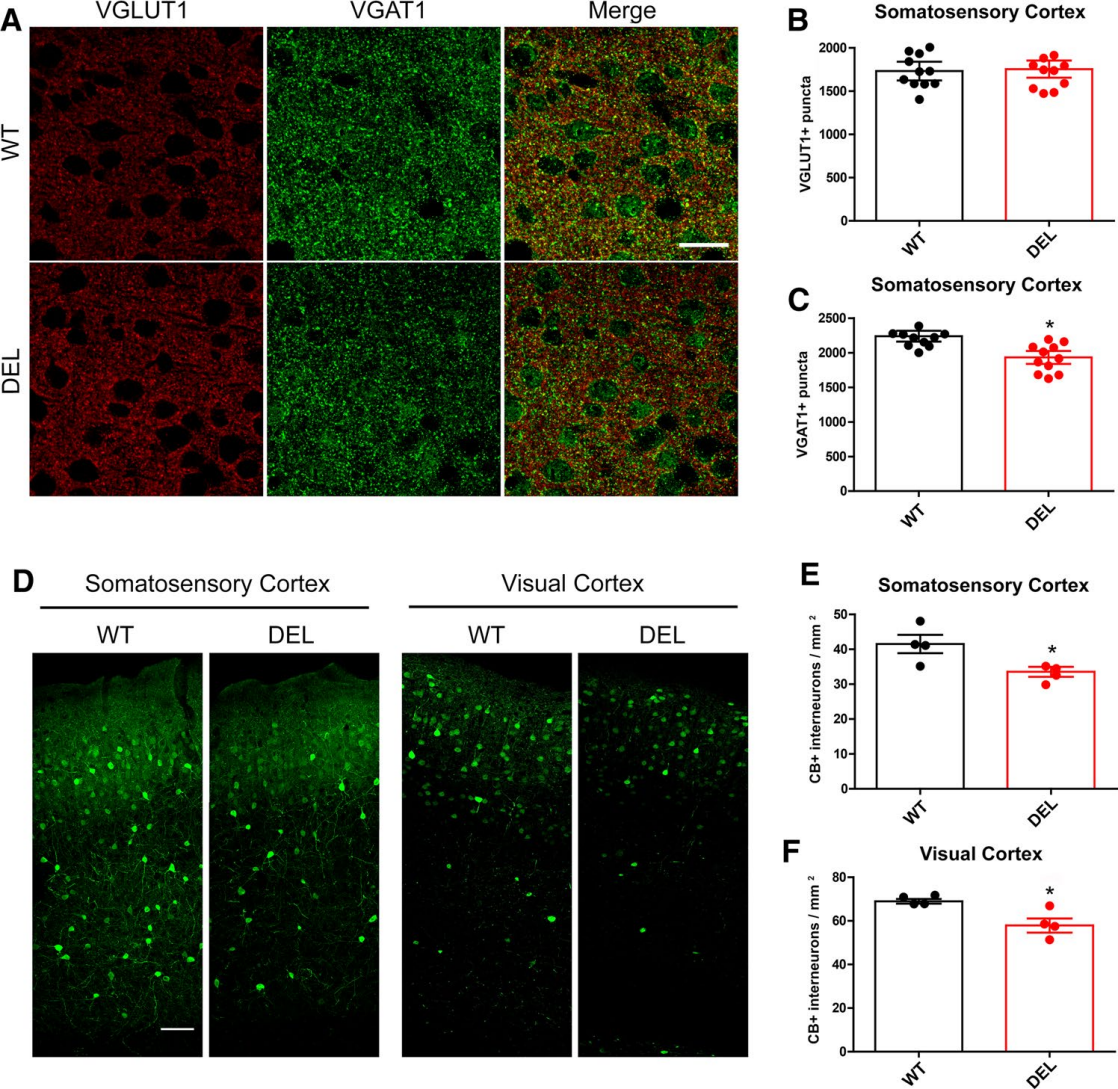
*Ncoa7* deletion affects inhibitory synapse density and alters the number of calbindin-positive interneurons in vivo*.* **a** Representative confocal images of the layer 2/3 in the somatosensory cortex of WT and DEL brain sections immunostained with VGLUT1, VGAT and their merged signals. Quantification of the number of VGLUT1 (**b**) and VGAT (**c**) immunoreactive puncta. **d** Representative calbindin immunostaining of the somatosensory and visual cortex from WT and DEL brain sections and **e**, **f** quantification. Data represents anatomically matched brain slices from *n* = 4 WT and DEL mice. Data are expressed as the mean ± SEM. *p < 0.05; unpaired Student’s *t* test

Next, we went on to analyse similar synaptic connections in WT and DEL mice in vivo using brain tissue by quantitative immunofluorescence of excitatory (VGLUT1) and inhibitory VGAT-positive synapses. While no differences in VGLUT1-positive puncta were found in the somatosensory cortex between WT and DEL mice (Fig. 7a, b), we found a significant decrease of VGAT-positive puncta in the same region in mutant animals (Fig. 7a, c). To complement these findings, we also assessed GABAergic interneurons, focussing on the calbindin (CB)-positive subtype that target dendritic compartments of other GABAergic neurons and pyramidal cells [48, 49, 50, 51]. Interestingly, the number of CB-positive interneurons was significantly reduced in the DEL somatosensory and visual cortex (Fig. 7d–f). Overall, these neuropathological results reveal an as-yet unreported role for NCOA7 in neuronal development, synaptogenesis and formation of cortical inhibitory circuits in vivo.

### *Ncoa7* deletion causes anxiety and social impairments in mice

Constitutive homozygous mouse knockout models of the TLDc genes *Oxr1* and *Tbc1d24* result in severe phenotypes that preclude the analysis of total loss-of-function in the context of adult behaviour [30, 51]. The viability of our new DEL model thus allowed us to investigate whether the absence of *Ncoa7* would cause behavioural alterations relevant to the gene’s known links to autism [27], or other features observed in TLDc gene rodent models, such as ataxia [30]. Moreover, lysosomal dysfunction is implicated in a range of neurobehavioual disorders, including those classified by anxiety and intellectual disability [5–10]. Therefore, we performed a battery of behavioural tests on adult WT and DEL mice.

**Fig. 8:**
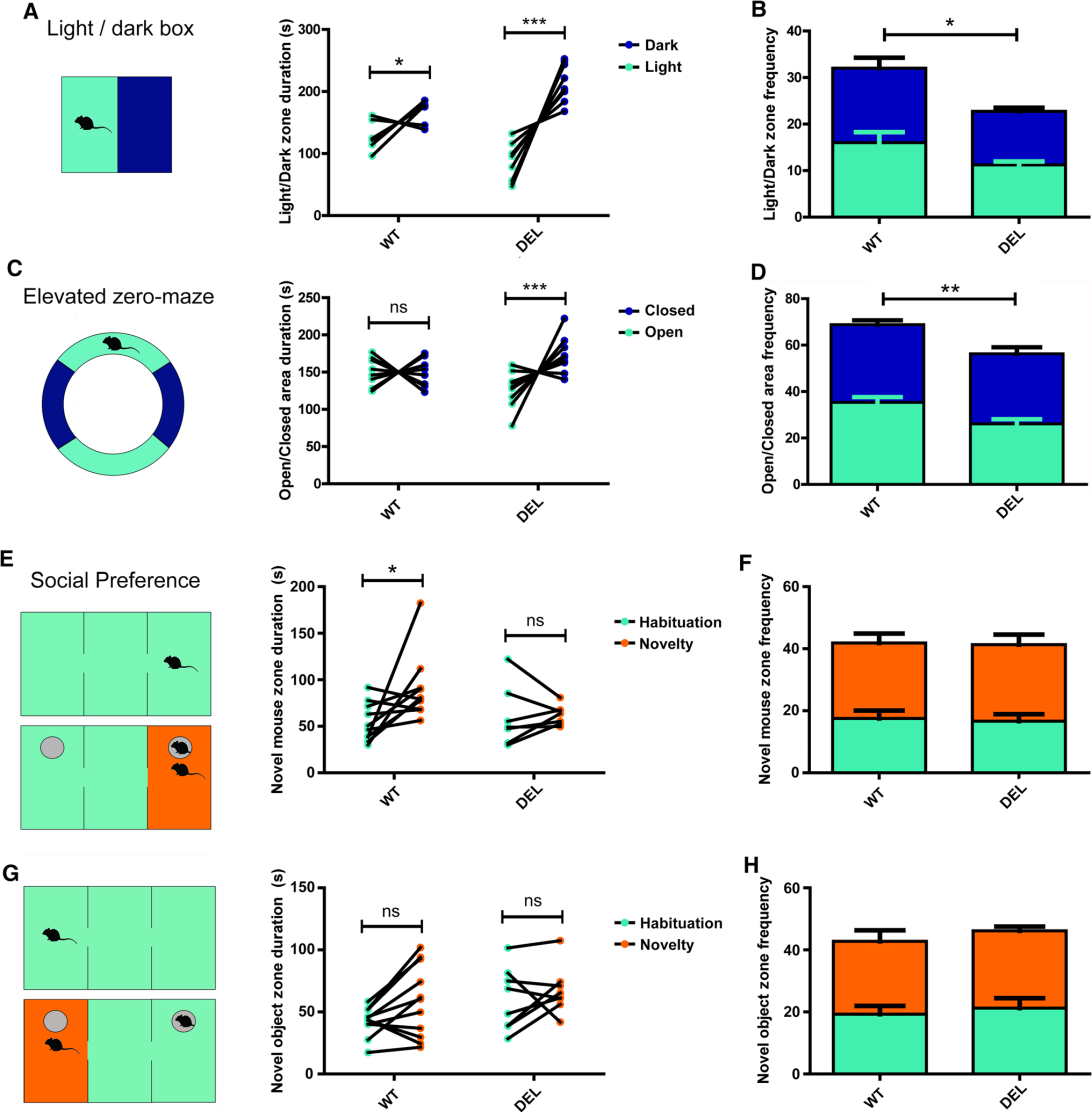
*Ncoa7* deletion induces anxiety-like behaviour and social impairment. **a** Light–dark box behavioural data plotted as total time in the light (green) or dark (blue) zones per WT or DEL animal during the trial and **b** the cumulative number of transitions between the light and dark (green) or dark and light (blue) zones. **c** Elevated zero-maze behavioural data plotted as total time in the light (green) or dark (blue) zones per WT or DEL animal during the trial and **d** frequency of entries into each zone. **e**–**h** Social preference testing during and initial habitation phase (green) and novelty phase (orange), where a new mouse in a cage is added to the apparatus (**e**) verses an empty cage (**g**). Data are plotted as zone duration (**e**, **g**) in addition to cumulative zone entrance frequency during both phases (**f**, **h**). *p < 0.05, **p < 0.01, ***p < 0.001, *ns *not significant. Two-way ANOVA/Bonferroni’s multiple comparison test. Data are expressed as the mean ± SEM. Data points represent individual animals

As an initial assessment of anxiety-related behaviour we used the light/dark box test. These data revealed a preference for DEL mice to remain in the dark and to make fewer visits between the light and the dark arenas compared to WT controls (Fig. 8a, b). These findings were consistent with additional data from the elevated zero-maze, where DEL mice spent more time in the closed area and entered the open area less frequently compared to WT littermates (Fig. 8c, d). Given that the interpretation of such tests can be confounded by motor impairment, we decided to analyse spontaneous activity and motor co-ordination using the open field and rotarod tests, respectively. The open field did not reveal any differences in mean velocity or total distance travelled between WT and DEL mice (Supplementary Figure S5A-B) and both genotypes performed equally well on the rotarod (Supplementary Figure S7C). These data indicate that loss of *Ncoa7* results in anxiolytic behaviour in mice without affecting motor capacity. Next, we examined aspects of cognition and memory by studying spatial novelty preference in a Y-maze and cued/contextual fear conditioning. Mice of both genotypes showed similar exploratory patterns during the habituation period and, above-chance preference for the novel arm in the Y-maze (Supplementary Figure S7D–G). During fear conditioning, while a decrease of the freezing episodes was observed during the training in DEL mice when compared to WT (Supplementary Figure S7H), no differences were observed in the freezing time during the training (Supplementary Figure S7I) or in any of the parameters analysed during both cue and context fear conditioning between the two genotypes (Supplementary Figure S7J–M). These data suggest that DEL mice are not significantly impaired in short-term memory acquisition and recall.

We also used the three-chamber test to analyse social-related behaviours in DEL mice. As observed in both the open field and Y-maze, no differences in the overall distance travelled were observed between genotypes during the task (Supplementary Figure S7N). Interestingly, we found that DEL mice spent less time with the novel mouse in the social novelty aspect of the test compared to the control WT littermates (Fig. 8e), although there were no differences frequency of visits between chambers (Fig. 8f). Furthermore, no differences were found in the time spent with the novel object, nor the frequency of visit to the novel object between the two genotype (Fig. 8g–h), suggesting that the previous observation was specific to interactions with another mouse. To test that this social impairment was not a result of submissive behaviour in DEL mice, we carried out the tube social dominance test, and these data suggested the level of submission/dominance was identical between the two genotypes (Supplementary Figure S7O). These results indicate that *Ncoa7* deletion results in impaired social behaviours in mice.

### One functional allele of either *Ncoa7* or *Oxr1* is required for viability in mice

**Fig. 9:**
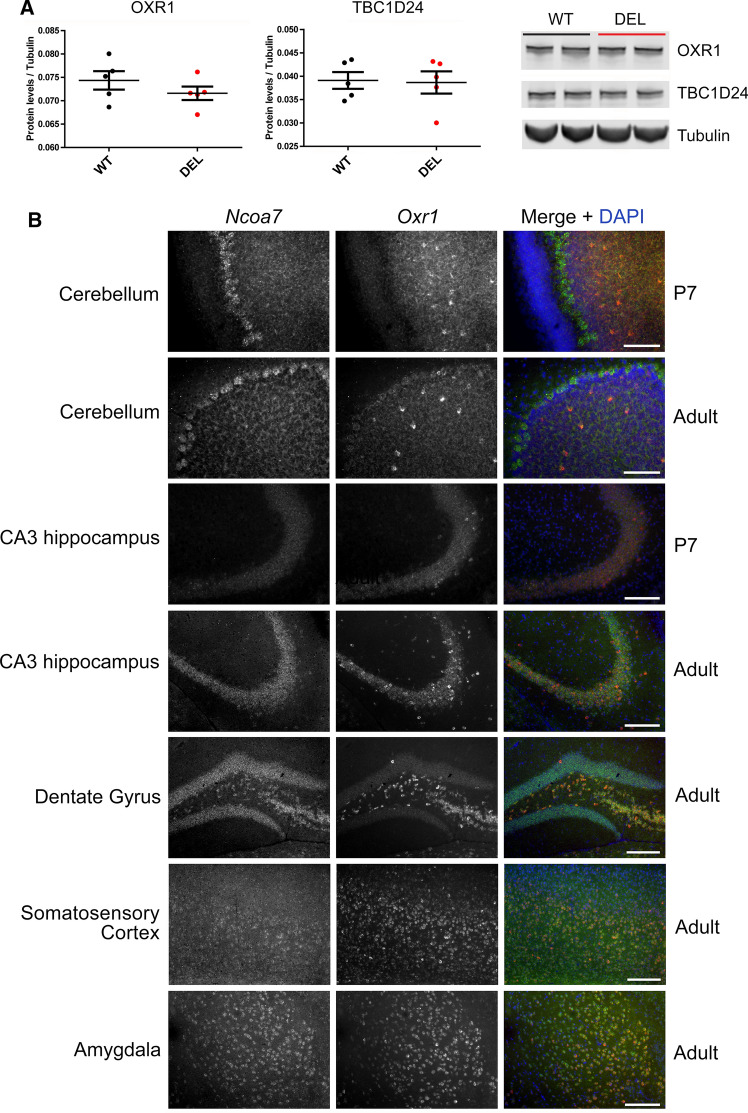
Expression of TLDc proteins in DEL mice and co-expression of *Oxr1* and *Ncoa7* in the brain. **a** Representative Western blots of TBC1D24 and OXR1 from the age-matched WT and DEL whole cerebral cortex tissue quantified with β3-tubulin as the loading control. **b** Dual-fluorescence in situ hybridisation of *Ncoa7* (green) and *Oxr1* (red) with the merged signal in the brain regions and timepoints indicated. Scale bars: 100 μm for cerebellum and 200 μm for the remaining panels. Data are expressed as the mean ± SEM. *n* = 5 for WT and DEL in (**a**)

The lack of an overt neurodegenerative phenotype in DEL mice was somewhat surprising considering the data from other TLDc rodent knockout models [30, 52]. We reasoned that this might reflect functional compensation by other TLDc proteins in the brain in response to a lack of NCOA7, and in particular OXR1 as the closest family member with the identical composition of protein domains [23]. However, Western blots from adult brain showed no differences in either OXR1 or TBC1D24 at the protein level between DEL and WT mice (Fig. 9a). Next, we carried out further expression analysis for *Oxr1* and *Ncoa7* in the brain to determine, where there may be detectable regions of co-expression. Indeed, there are many regions of the brain, where cells express both genes at a high level, including the motor cortex and hippocampus (Supplementary Figure S1). In the cerebellum, however, a generally reciprocal expression pattern is observed with *Oxr1* expressed predominantly in the granule cell layer, whereas *Ncoa7* is highly enriched in Purkinje cells (Fig. 9b and Supplementary Figure S1). From these data, despite no evidence for an induction of expression, we hypothesise that *Oxr1* may be able to compensate for a total lack of all *Ncoa7* isoforms in our DEL model.

To investigate further the requirement for *Oxr1* and *Ncoa7 *in vivo, we decided to cross the NCOA7 DEL mutants to our previously described constitutive *Oxr1* knockout model in which all isoforms of the gene are disrupted (*Oxr1*^*d/d*^) [53]. We discovered that mice carrying only one functional allele of either *Ncoa7* or *Oxr1* were viable and were born at the expected Mendelian ratios (Supplementary Table S1). In addition, while *Oxr1*^*d/*+^/*Ncoa7*^*del/del*^ mice did not show any signs of cell death in the brain up to 18 months of age (data not shown), *Oxr1*^*d/d*^*/Ncoa7* heterozygous DEL knockout (*Ncoa7*^*del/*+^) mutants showed the same phenotypic pattern of early-onset ataxia as *Oxr1*^*d/d*^ animals. Thus, to ascertain whether the distinctive neuropathology of *Oxr1*^*d/d*^ mice was modified by the loss of one *Ncoa7* allele, we quantified cell death in cerebellar sections at postnatal day (P)22; these data indicated that there was no significant difference in the severity of neurodegeneration between *Oxr1*^*d/d*^/*Ncoa7*^*del/*+^ and *Oxr1*^*d/d*^*/Ncoa7*^+*/*+^ mice (Supplementary Figure S8).

Interestingly, from all the breeding carried out, no live double knockouts (*Oxr1*^*d/d*^/*Ncoa7*^*del/del*^) were born (Supplementary Table S1), suggesting that mice of this genotype are not viable. We also carried out genotyping of E8.5–9.5 embryos from the same genetic cross and again no double homozygous knockout genotypes were obtained (Supplementary Table S1). Together, these data show for the first time the essential requirement for at least one functional allele of *Ncoa7* or *Oxr1* for embryonic development and post-natal viability and emphasises the functional importance of these two TLDc proteins in vivo.

## Discussion

Here we demonstrate for the first time in vivo that NCOA7 binds to the cytoplasmic V1 domain of the V-ATPase and that it also modulates the assembly and functionality of this essential proton pump in the brain. Importantly, we could also rescue the defects in lysosomal homeostasis we observed caused by deletion of *Ncoa7* by exogenous replacement of the protein. We show an interaction of NCOA7 with several subunits critical for the correct formation of the V-ATPase V1 complex domain, including ATP6V1B2 and ATP6V1C [54]. Indeed, variants in human ATP6V1B2 that are predicted to alter interactions within the V1 complex cause dominantly-inherited neurodevelopmental disorders [55, 56]. Although we observed a significant shift in distribution of the ATP6V1B2 and ATP6V1A1 subunits away from the membrane in DEL neurons, no changes in the absolute levels of ATP6V1B2 were detected in the brain or primary cells. These data are consistent with a number of other crucial V-ATPase modulators—such as the accessory protein ATP6AP2—that does not affect subunit expression, but does disturb the ratio of membrane-assembled ATP6V1B2 to V0 subunits [55, 56]. Of note, de novo ATP6AP2 deletion variants have also been described in neurological disease, altering neuronal development, LC3b abundance and lysosomal function when modelled in the mouse and human stem cell-derived neurons [56, 57]. Furthermore, absence of VPS50, another V-ATPase accessory protein, not only alters dense-core vesicle acidification, but is also implicated in neural tube formation in humans [58]. Thus, our data provides further evidence for the importance of V-ATPase regulation for neurodevelopment. Moreover, our detection of cytosolic accumulation of potentially undegraded material in DEL neurons is consistent with similar examples of V-ATPase subunit disruption [59, 60], supporting our findings that the absence of NCOA7 negatively affects lysosomal physiology. We did not, however, observe evidence for more overt lysosomal storage in primary neurons as has been described in other V-ATPase-associated mutant models [56].

We further demonstrate that loss of *Ncoa7* causes altered dendritic outgrowth, a reduction in the number of CB interneurons and the numbers of inhibitory synapses; in addition, homozygous mutants exhibited anxiolytic behaviour in two behavioural assays and altered sociability in the three-chamber test. Multiple brain structures are involved in the regulation of anxiety and social behaviours, such as the amygdala, thalamus, hippocampus and cortical areas [61–65]. Our developmental expression data show that *Ncoa7* is found in all of these key neuroanatomical regions, in addition to those related to olfaction, a fundamental feature of mouse exploratory activity, anxiety and sociability [62]. Furthermore, direct alterations of excitatory and inhibitory balance within the brain have a strong effect on social motivation, potentially underlying the deficits seen in social novelty and interaction [66]. Deficient GABAergic transmission has been described in anxiety [67, 68], and enhancement of GABAergic transmission through pharmacological action has been shown to reduce anxiety behaviours [69, 70]. Conversely, GAD67, the enzyme responsible for GABA production, has been linked to anxiety disorders [71]. We postulate that the behavioural effects of *Ncoa7* deletion we see may result from the reduced CB-positive interneurons in the cortex and the subsequently reduced number of inhibitory synapses observed in the cerebral cortex. This overall reduction of the inhibitory circuitry observed in *Ncoa7* mutant mice may affect the connectivity between cortical and ventral brain areas involved in the regulation of anxiety and sociability, behavioural outcomes relevant to the recent association of the gene to autism [27].

Our cellular localisation studies point towards a membrane-associated role for NCOA7, including interactions with a number of specific PIPs that are not only implicated in vesicle trafficking and endolysosomal homeostasis, but also the recruitment of the V-ATPase to the membrane [37–39, 72]; indeed, the range of PIP interactions suggest multiple potential intracellular sites of action. Interestingly, structural studies have demonstrated that the TLDc protein TBC1D24 contains a PIP-binding pocket situated in the N-terminal TBC domain [73], thus it is noteworthy that the same repertoire of PIPs interact with both TBC1D24 and NCOA7 [73]. Furthermore, it is particularly intriguing that in a recently described microdeletion syndrome, certain clinical features including seizures have been narrowed-down genetically to haploinsufficiency of *TBC1D24* and the neighbouring V-ATPase V0 subunit gene *ATP6V0C* [74, 75]. Together with the description of OXR1 as a potential ATP6V1B1 interactor in kidney tissue [32] and lysosomal abnormalities reported in cells derived from loss-of-function *OXR1* patients [17], it is apparent that the TLDc domain may be a critical functional regulator of V-ATPase function, with alternative domains (e.g., TBC or GRAM) assisting with anchoring to the membrane.

One reason to generate this unique *Ncoa7* deletion model was in part due to the presence of the short TLDc domain-containing isoform of NCOA7 (NCOA7-B), considering that we and others have shown that a similar short OXR1 isoform can compensate for disruption of the corresponding full-length version [29, 30, 53]. In a very recent example, disruption of a single, full-length, brain-specific OXR1 isoform in the mouse resulted in growth hormone release abnormalities [76], but no neurodegeneration as we observe in our *Oxr1*^*d/d*^ knockout [30]. Therefore, we might have expected a more severe phenotype in the homozygous DEL mutant described here that lacks all possible *Ncoa7* isoforms. Specific activation of the short NCOA7-B isoform occurs via an interferon (IFN)-mediated pathway and has been shown to be protective against viral infection in vitro [29, 77]. It is, therefore, possible that the brain does not require this particular short isoform to be active under basal conditions, and the presence of OXR1 in *Ncoa7* DEL animals is sufficient to avoid the severe neurodegenerative phenotypes caused by complete OXR1 loss-of-function as observed in humans and mice [17, 30].

Our data suggest that aspects of endolysosomal dysfunction caused by loss of all *Ncoa7* isoforms in the nervous system may underlie the behavioural changes we observe, although additional roles of the gene—including those yet to be determined—may also be involved. NCOA7 is classified as an ER-mediated transcriptional regulator, even if experimental evidence for this function is still limited. Yet it is noteworthy that other steroid receptor coactivators such as NCOA1/SRC1 are known to be important, rate-limiting modulators of transcriptional cascades implicated in neurodevelopment and behaviour, including aspects of cognition in Alzheimer’s disease [78]. Furthermore, with specific relevance to the recent discovery of *NCOA7* mutations in autism, several de novo genetic variants in the nuclear receptor corepressors *NCOR1* and *NCOR2* are found in pediatric patients with intellectual disabilities or ASD [78]. As such, despite our fractionation experiments in brain tissue failing to detect NCOA7 in the nucleus, the deregulation of neurodevelopmentally-critical transcriptional pathways due to a deletion of *Ncoa7* cannot be ruled-out.

In the kidneys of DEL mice a significant reduction in ATP6V1B1 expression was observed, similar to studies of an *Ncoa7* full-length isoform mutant (*Ncoa7-tm1.1*) [33]. We were also able to show in vivo binding between NCOA7 and ATP6V1B1, yet the cause of this reduced expression—not observed for V1 subunits in the brain from our DEL mutant—is unclear. It is possible that destabilised V-ATPase subunits are targeted for degradation; indeed, a chaperone-like role for NCOA7 binding to the B1 subunit is plausible, given the similar role for OXR1 in stabilising catalytic multimers [79, 80]. Alternatively, a tissue-specific transcriptional feedback loop involving the regulatory activity of NCOA7 on V-ATPase subunit expression could be involved. Importantly, our urine pH data are consistent with V-ATPase dysfunction in the intercalated cells of the kidney as well as an incomplete renal acidosis (dRTA) phenotype as described in *Ncoa7-tm1.1* homozygous mutants and *Atp6v1b1* knockout mice [33, 81]. The increase in urine pH of approximately 1 unit in DEL mice is comparable to that in the homozygous *Ncoa7-tm1.1* line [33], suggesting that the additional removal of the short NCOA7-B isoform in DEL mutants does not exacerbate V-ATPase dysfunction in the kidney. Undoubtedly, further functional studies of both NCOA7 isoforms outside of the nervous system are warranted in the future.

Our in situ hybridisation studies of *Ncoa7* and *Oxr1* presented here show obvious regions of co-expression across the brain, although there was no evidence for the induction of OXR1 or TBC1D24 at the protein level in *Ncoa7* DEL tissue. It is particularly interesting in the mouse cerebellum that *Oxr1* is expressed predominantly in the granule cell layer of neonates and adults, whereas *Ncoa7* is clearly enriched in Purkinje cells at both timepoints. These data suggest that the specific pattern of cell death apparent in the granule cell layer of *Oxr1*^*d/d*^ animals may be due to a relative lack of *Ncoa7* that is unable to compensate functionally compared to other brain regions at that timepoint. To begin, therefore, to assess a genetic interaction between TLDc proteins for the first time in the mouse in vivo, we crossed our DEL mutant with an *Oxr1* loss-of-function model such that the TLDc domain of all potential isoforms of both genes could be disrupted. Although mice haploinsufficent for *Oxr1* on an *Ncoa7* knockout background and those heterozygous for *Ncoa7* in the *Oxr1* knockout model are viable, no double-null mutants were obtained even at early embryonic timepoints. This underlies the importance of TLDc genes during development and demonstrates that not all proteins that contain this conserved domain can fully compensate for one another in a mammalian system; future work will assess in more detail the genetic interaction between TLDc family members. Indeed, loss-of-function mutations of ubiquitously-expressed V-ATPase subunits are non-viable in mice, where there is no functional redundancy in the system [82].

In summary, mice lacking the entire *Ncoa7* locus lead to altered V-ATPase formation in neurons alongside lysosomal defects that influence neuronal development, synaptic formation and ultimately behaviour. It has become clear that the V-ATPase has profound effects on many pH-dependent cellular processes [83]; consequently, regulation of V-ATPase assembly and function has been proposed as a viable therapeutic target in a wide range of human disorders [84–86]. In the future, it will be important to determine whether TLDc proteins can be harnessed as a novel V-ATPase gating mechanism applicable to many physiological states, where there is a fundamental necessity to control vesicular acidification, not limited to the obvious importance of this process for neuronal development and connectivity.

## Materials and methods

### Experimental animals and housing

Animals were generated and maintained under UK Home Office Project Licences 30/2966, 30/3353 and P7133CD66 with local ethical guidelines issued by the University of Oxford and the Medical Research Council Harwell Institute. Animals had ad libitum access to food and water and were kept under a controlled 12 h light/dark cycle, temperature (21 ± 2 °C) and humidity (55 ± 10%). All experimental animals were maintained on a C57BL6/J background and backcrossed over 10 generations before intercrossing, eliminating the risk of any residual off-target mutagenesis contributing to the phenotype. The *Ncoa7* deletion (DEL) line was created by CRISPR/Cas9-mediated deletion of entire *Ncoa7* coding region. Guide-RNAs were designed using the CRISPOR algorithm (http://crispor.tefor.net) against genomic sequences lying immediately upstream and downstream of the *Ncoa7* start and stop codons and selected on the basis of genome specificity, with at least 3 mismatches in distal positions relative to the PAM needed to achieve an off-target match against the mouse genome. Guide-RNAs were designed and validated as described previously [19], but here for activity against genomic sequences lying immediately upstream and downstream of the *Ncoa7* start and stop codons (#740: 5′-GCGCCCGGCGCCCTGACCGT-3′ and #736: 5′-ACATGAGTTGGCGAGATCCC-3′). The guides were generated by in vitro transcription and microinjected at a concentration of 5 ng/μl, together with Cas9 mRNA at 10 ng/μl, into a pronucleus of fertilized C57BL/6J oocytes. In the resulting litters, a founder mouse was identified with a confirmed deletion spanning 157,481 bp that was successfully transmitted to the next generation. Breakpoints were confirmed by Sanger sequencing. Mice were screened initially using primers for presence of the deletion: Ncoa7-F1 5′-AGGCAATGCTGGAAATGACAGAGG-3′ (wild-type penultimate coding exon), Ncoa7-R1 5′-CCCTGGAATGGATCATGTCATGTC-3′ (wild-type 3′ UTR sequence and 3′ end of deletion) and Ncoa7-F2 5′-GATTGCCTCCCTCTTATGAAAGCC-3′ (wild-type 5′UTR sequence and 5′ end of the deletion). *Oxr1* knockout animals were genotyped as described previously [53].

### Behavioural phenotyping tests

For all behavioural phenotyping tests, mice were taken to the test room at least 20 min before the start of the test to acclimatise and apparatus was cleaned between individuals to avoid olfactory cues. Investigators were blind to genotype during all phenotyping tests. A battery of tests was carried out on cohort of age-matched female WT (*N* = 8) and DEL (*N* = 9) starting at 3 months of age in the order described below. For video tracking, all experiments were analysed using Ethovision XT software (Noldus, The Netherlands).

#### Open field activity

Animals were placed individually in square opaque Plexiglas arenas (44 × 44 cm) with lighting set at 150–200 lux and were video-tracked for 20 min.

#### Elevated zero-maze

Mice were placed in an open arm of an elevated ring-shaped runway with the same area devoted to adjacent open (30 cm × 5 cm) and closed quadrants (30 cm × 5 cm × 15 cm) and video-tracked for 5 min, during which their preference for the open or closed quadrant was scored.

#### Dark–light box

Mice were placed in the open area of the box consisting of an open (40 cm × 40 cm × 20 cm) and closed (40 cm × 20 cm × 40 cm) area and video-tracked for 10 min, during which their preference for the open or closed area was scored.

#### Y-maze

To evaluate short-term working memory mice were placed in the ‘start’ arm of a Y-shaped transparent maze (three identical arms 35 cm × 5 cm × 15 cm) with access to one arm blocked, they were then free to explore the ‘start’ arm and the ‘familiar’ arm for 10 min. Mice were returned to the home cage for a two-minute inter-trial interval, and then returned to the maze, with access to all three arms for 5 min with time spent in the ‘novel’ versus ‘familiar’ arm recorded.

#### Rotarod

To assess coordination and motor learning, mice were placed on an accelerating rotarod (Ugo Basile), with rotor speed increasing from 4 rpm up to 40 rpm over 5 min. The time taken for the mouse to fall from the rod was recorded. One trial per day was carried out over three consecutive days.

#### Contextual and cue fear conditioning

Conditioning phase: a mouse was placed in the test chamber [Fear Conditioning System (Ugo Basile, Italy)] and allowed to explore freely for 2 min. A pure tone (5 kHz, 80 dB) serving as the conditioning stimulus (CS) was played for 20 s. During the last 1 second of the tone, a foot shock (0.5 mA) was delivered as the unconditioned stimulus (US). The mouse was left in the chamber for another 2 min. Context phase: 24 h after conditioning, mice were placed in the same chamber and freezing behaviour recorded for 5 min in the absence of any stimuli. Mice were then returned to their home-cage. Cue phase: 4 h after the context phase, mice are placed in a round Plexiglas cylinder with vanilla fragrance spread at the top of the cylinder for 6 min during which the mouse is exposed to the same sound as in the conditioning phase. Both freezing behaviour and activity was scored.

#### Three chamber social test

Mice were placed in a middle chamber (23 cm × 40 cm × 22 cm) of a transparent arena with access to both of the other two chambers (23 cm × 40 cm × 22 cm) via an opening. They were then free to explore the arena for ten minutes. Mice were returned to the home cage for a two-minute inter-trial interval, during which the arena was cleaned to remove odour cues, and an object (single-mouse cage) and a novel mouse (in a single-mouse cage) were placed in either the left or right chamber, during which their preference for the object or the novel mouse was scored by the time spent and the frequency of visits in the novel object’s or novel mouse’s chamber. The place of the object and the novel mouse were alternated during each trial.

#### Social dominance test

One age-matched WT and DEL mouse of a similar wight were allowed to enter at opposite ends of a transparent polyvinyl chloride tube (30 cm × 4 cm). After the mice meet at the centre of the tube, the first mouse to reverse out of the tube is considered subordinate. Each mouse performed 5 challenges against a different mouse and the percentage of challenges won was recorded.

### Primary neuronal cultures and transfection

Low-density primary cortical neurons were prepared from P0 brains as previously described [86]. Briefly, cerebral cortices were dissociated by enzymatic digestion and dissociated neurons were plated at low density (200 cells/mm^2^) onto poly-d-lysine-coated 25 mm glass coverslips. For evaluation of rescue experiments, DEL neurons were nucleofected before plating with the 4D-Nucleofector System (Lonza) with the high viability protocol for primary mouse neurons and then analysed for GFP expression. Mouse full-length NCOA7 constructs were either C-terminal HA-tagged in vector pcDNA3.1 or C-terminal GFP-tagged in vector pEGFP.

### Real-time PCR

Total RNA was isolated from different brain areas as previously described [86] and 500 ng used for cDNA synthesis with RevertAid reverse transcriptase (Thermofisher Scientific). Real-time PCR analyses were performed using SYBR Green I Mastermix (Roche) on a 7500 Fast Real-Time System (Applied Biosystem) with the following protocol: 95 °C for 10 min; 30 s at 95 °C/20 s at the specific annealing temperature (Ta)/30 s at 72 °C for 45 cycles; melting curve (heating ramp from 55 to 95 °C) to check for amplification specificity. Each individual well used 1/100 of the original cDNA reaction. The *Ncoa7 *exon 3-specific primers FW 5′-CAGCTGCGTCCTCGAAGAG-3′ and Rev 5′-GCTGTAACGTTGAACTTGTCTTGTTC-3′ were used to detect *Ncoa7* full-length transcripts and S16 FW 5′-TTCTGGGCAAGGAGCGATT-3′ and Rev 5′-GATGGACTGTCGGATGGCA-3′ was used as internal control.

### Subcellular fractionation, immunoprecipitation and western blotting

Low density cortical primary neurons were plated as described above. Neurons were placed on ice and washed twice with ice-cold PBS. Neurons were scraped into homogenization buffer (250 mM sucrose, 1 mM EDTA, 10 mM PMSF, 2 μg/ml aprotinin, 5 μg/ml leupeptin, and 1 μg/ml pepstatin) and lysed by repeated passage through a ball bearing homogenizer. The homogenate was cleared of intact cells and nuclei by centrifugation at 500×*g* for 10 min. The supernatant was subjected to ultracentrifugation at 100,000×*g* for 30 min to pellet the membrane fraction. The supernatant containing the cytosolic fraction was concentrated using Amicon Ultra 10 K centrifugal filters according to the instructions of the manufacturer. Resulting cytosolic samples were supplemented with 1% SDS to yield the final cytosolic fractions. Membrane pellets were rinsed once with homogenization buffer and resuspended in homogenization buffer containing 1% SDS to yield the final membrane fractions. NCOA7, ATP6V1B2, ATP6V1A, and ATP6V1C were immunoprecipitated from cell lysates using the Pierce Crosslink Immunoprecipitation Kit (Thermo Fisher Scientific) as specified by the manufacturer. Antibodies were coupled to the Pierce Protein A/G Plus Agarose for 1 h, and then crosslinked to the resin using the DSS crosslinker (2.5 mM in DMSO) for 1 h. Subsequently, 500–1000 µg of lysates were incubated on the column overnight at 4 °C. The eluates were finally loaded on NuPAGE Bis–Tris 4–12% polyacrylamide precast gel and analysed for presence of the antigen. Western blotting experiments and analysis were performed as previously described [87]. Primary neurons and brain tissue were lysed in RIPA buffer using Lysing Matrix tubes for homogenisation (MP Biomedical). The following antibodies were used: anti-NCOA7 (1:1000, ProteinTech 23092-1-AP), anti-ATP6V1B2 (1:2000, ProteinTech 15097-1-AP), anti-ATP6V1A(1) (1:2000, ProteinTech 17115-1-AP), anti-ATP6V1D(1) (1:1000, ProteinTech 14920-1-AP), anti-ATP6V0a1 (1:1000, ProteinTech 13828-1-AP), anti-ATP6V0d1 (1:1000, ProteinTech 18274-1-AP), anti-AT1A1 (1:1000, Millipore 05-369), anti-viniculin (1:500, Cell Signaling 4650), anti-ATP6V1B1 (1:1000, ProteinTech 14780-1-AP), anti-tubulin (1:5000, Biolegend 657401), anti-Cathepsin D (1:2000, Abcam ab6313), anti-Cathepsin B (1:1000, Merck ab125067), anti-LAMP1 (1:1000 Abcam ab24170), anti-Rab7 (1:1000, Abcam ab137029), anti-Cofilin (1:1000, Abcam ab42824), anti-TBC1D24 (1:1000, Proteintech 25254-1-AP) and anti-OXR1 (1:1000, Proteintech 13514-1-AP). Proteins was visualized using anti-mouse (P/N 926–68,070) or anti-rabbit (P/N 926–32,211) secondary antibodies IRDye (LiCor Biosciences) at 1:10,000 dilutions and quantified using the scanning infrared Odyssey imaging system CX (LiCor Biosciences).

### PIP interaction assay

Full-length NCOA7 recombinant protein (OriGene) was incubated with PIPs strip Membrane (ThermoFisher) containing 100 pmol of lipid per spot overnight. The membrane was then incubated with an anti-NCOA7 (1:1000, ProteinTech). The protein–lipid interaction was visualized using an anti-rabbit secondary antibody IRDye (LiCor Biosciences) at 1:10,000 dilution and quantified using the scanning infrared Odyssey imaging system CX (LiCor Biosciences).

### Immunofluorescence and immunohistochemistry

Immunofluorescence experiments and analysis were performed as previously described [84]. For Ncoa7 mutant tissue analysis, animals were transcardially perfused with 4% PFA, sections cut at 30 μm using a vibratome (Leica) and processed for free-floating immunofluorescence. The following primary antibodies were used: mouse ant-NeuN (1:500, Millipore MAB377), rabbit anti-VGAT (1:300, Synaptic Systems 131003), mouse anti-VGLUT1 (1:250, Synaptic Systems 135 311) and anti-Calbindin (1:5000, Novus Biological). Images were acquired with a Zeiss LSM 700 confocal microscope (Carl Zeiss AG) using the multitrack mode to avoid fluorescence crosstalk (pinhole: 1.0 airy unit). For cell death quantification in Oxr1 mutants, 14 μm parasagittal sections from perfused brains taken at P22 were immunostained for cleaved caspase-3 Asp175 (1:500, Cell Signaling) and the Vectorstain Elite ABC kit (Vectorlabs). A total of 8 anatomically matched sections at equidistant intervals from 4 mice of each genotype were visualized by light microscopy. For the quantitative analysis of VGAT and VGLUT1 puncta, fluorescence levels were analysed on confocal images by measuring pixel intensity using ImageJ. Histology was performed on neurons labelled with anti-NeuN. Frontal, medial and caudal sections were chosen to calculate cortical regions, corpus callous and hippocampal regions thicknesses. Images from the cerebellum were always acquired from the fourth/fifth lobe to measure the thickness of granule cell and molecular layers. Images were acquired at 20× and all parameters were analysed using Fiji software. Immunofluorescence on primary neurons was performed as previously described [88]. Briefly, neurons were fixed in 4% paraformaldehyde and then immunostained. Fluorescence intensity and density of positive-puncta measurements were performed using ImageJ. Fluorescence intensity of endogenous LAMP1 staining was measured as mean intensity and expressed in arbitrary units of fluorescence per cell area. LAMP1 positive puncta were measured in each z-stack and normalised on the cell area. Antibodies were used as follows: anti-LAMP1 (1:500; Developmental Studies Hybridoma Bank 1D4B), anti-β3-tubulin (1:1000; Biolegend), anti-GFP (1:1000; Thermo Fisher Scientific A-11122). As a control for the staining and the acquisition procedure, the primary antibodies were omitted. The analysis of synapse density was performed as previously described [43]. Sholl analysis was performed as previously described [18], and the number of intersections was automatically evaluated with the ImageJ/Sholl analysis plug-in.

### Live imaging

For LysoTracker Green DND-26 (Thermo Fisher Scientific L7526 ) experiments, primary cortical neurons (7-DIV) were incubated with LysoTracker solution (LysoTracker 100 nM in Neurobasal-A (Thermo Fisher Scientific) complemented with 1% Penicillin–Streptomycin, 1% GlutaMax, 2% B-27 (Thermo Fisher Scientific) for 10 min at 37 °C and 5% of CO_2_. At least 20 neurons each from 3 different preparation were imaged. After incubation, neurons were washed with FluoroBrite imaging media (Thermo Fisher Scientific) and immediately acquired with a Zeiss LSM 700 confocal microscope (Carl Zeiss AG) equipped with a 37 °C and 5% CO_2_ chamber. For the quantitative analysis of LysoTracker-positive organelles, Z-series stacks of 4 consecutive confocal Sections (1024 × 1024) were acquired at 40× magnification. Images were acquired in less than 20 min to prevent LysoTracker dispersion. Lysotracker positive puncta were measured in each z-stack and normalised on the cell area. To determine the degradation of ectopically transduced dextran, primary cortical neurons (7–10 DIV) were incubated with 100 μg/ml Alexa Fluor 594 labelled dextran (Thermo Fisher Scientific D22913) for 6 h. After washing with Tyrode solution (140 mM NaCl, 3 mM KCl, 2 mM CaCl_2_, 1 mM MgCl_2_, 10 mM glucose, 10 mM HEPES-buffered to pH 7.4), neurons were immediately imaged with a Zeiss LSM 700 confocal microscope (Carl Zeiss AG) equipped with a 37 °C and 5% CO_2_ chamber, or incubated with fresh Neurobasal-A complemented as described above for 1 h and then acquired. To live label protease-mediated hydrolysis of BSA, primary cortical neurons (7-DIV) were incubated with 50 μg/ml DQTM Red-BSA (Thermo Fisher Scientific D12051) at 37 °C for 2 h. Neurons were then washed with Tyrode solution and immediately live imaged as described above, or incubated with fresh Neurobasal-A complemented as described for 1 h and then acquired. All the parameters were analysed using Fiji software. Fluorescence intensity of the different dyes was measured as mean intensity and expressed in arbitrary units of fluorescence on the cell area.

### FITC-dextran pH calculation

To determine the pH of endocytic compartments, primary cortical neurons were incubated with 100 μg/ml of fluorescein isothiocyanate (FITC) labelled dextran (Merck Millipore 46944) for 2 h. After washing with Neurobasal medium, supplemented as described above, cells were incubated with fresh medium for 30 min. Neurons were live imaged at the confocal microscope as described above. A pH calibration curve was then collected for each coverslip using 10 μM Valinomycin and 10 μM Nigerin in HEPES buffer (pH 7.5) and MES buffer (125 mM KCl, 25 mM NaCl, 25 mM MES, pH 6.5, 5.5 and 4.5). FITC-Dextran intensity in both channels was calculated after background subtraction and ratio (495/450) were fitted to a linear regression with the GraphPad Prism 5 software. Data were best fitted in the range of pH 4.5–7.5. For pH evaluation, measured ratios were converted into absolute pH values by interpolation in the calibration function (*y* = 31.751x–138.76, *R*^2^ = 0.9873).

### In situ hybridization

For riboprobe synthesis, regions representing all isoforms of *Oxr1* (3085–3486 bp of NCBI accession number NM_001130166.1) and *Ncoa7* (3261–3572 bp of NM172495.6) were used. DIG-labelled non-fluorescent probe hybridisation and detection on fresh frozen Sections (14 μm) for *Ncoa7* was carried out as previously described [89]. For dual-fluorescent reactions, the *Oxr1* riboprobe was DNP-labelled and *Ncoa7* was DIG-labelled. Probe hybridization and detection was carried out using the TSA Plus Fluorescence Systems with Cy3 and Fluorescein as per the manufacturer’s instructions (Perkin Elmer), using sheep-anti-DIG-POD Fab fragments (1:500; Roche) or anti-DNP-POD (1:100; Perkin-Elmer) and a final 1:100 dilution of the tyramide detection reagent for 15 min. Peroxidase activity of the previous antibody was inactivated by washing the slides with PBS containing 0.1% Tween-20 and 6% H_2_O_2_ for 10 min prior to labelling with the second riboprobe.

### Electron microscopy

For transmission electron microscopy, low density primary cortical neurons were fixed at 14–18 DIV with 1.2% glutaraldehyde in 66 mM sodium cacodylate buffer, post-fixed in 1% OsO_4_, 1.5% K_4_Fe(CN)_6_, 0.1 M sodium cacodylate, en bloc stained with 1% uranyl acetate, dehydrated, and flat embedded in epoxy resin (TAAB Epon 812). After baking for 48 h, the glass coverslip was removed from the Epon block by thermal shock and neurons were identified by means of a stereomicroscope. Embedded neurons were then excised from the block, and mounted on a cured Epon block for sectioning using an EM UC6 ultramicrotome (Leica Microsystems). Ultrathin Sections (60–70 nm thick) were collected on 200-mesh copper grids (EMS) and observed with a FEI Tecnai T12 transmission electron microscope operating at 100 kV using an ORIUS SC1000 CCD camera (Gatan). For each experimental condition, at least 30 images were acquired at 10,000× magnification (sampled area per experimental condition: 20 µm^2^). Neuronal morphometry were determined using ImageJ software.

### Urine analysis

Mouse urine was collected directly onto Hydrion Urine & Saliva pH Paper 5.5–8.0 (Micro Essential Laboratory) between 07.30 and 08.30 am and pH was read by two different, blinded investigators.

### Statistical analysis

Data are expressed as the mean ± SEM throughout. To compare two normally distributed sample groups, the two-tailed Student’s t-test was used. In case of more than two normally distributed experimental groups, one- or two-way ANOVA followed by multiple comparison tests were employed as stated. Significance level was preset to *p* < 0.05. Data were analysed using Prism 6.0 (GraphPad Software, Inc.) software.

### Supplementary Information

Below is the link to the electronic supplementary material.

AUTHOR NOTE: The supplementary file 1 still contains the highlighted (in yellow) text from the resubmission that was added from the first submission. This needs to be converted to non-highlighted text before uploading pleaseSupplementary file1 (DOCX 22 KB)

Supplementary file2 (TIF 5259 KB)

Supplementary file3 (TIF 3119 KB)

Supplementary file4 (TIF 360 KB)

Supplementary file5 (TIF 802 KB)

Supplementary file6 (TIF 407 KB)

Supplementary file7 (TIF 618 KB)

Supplementary file8 (TIF 409 KB)

Supplementary file9 (TIF 9688 KB)

## Data Availability

Contact corresponding author.

## Abbreviations

ASD: Autism spectrum disorder
AT1A1: Sodium/potassium-transporting ATPase subunit alpha-1
CB: Calbindin
CNS: Central nervous system
ERAP40: Estrogen receptor (ER)-associated protein 140
GABA: Gamma-aminobutyric acid
GRAM: Rab-like GTPase activators and myotubularins
IFN: Interferon
NCOA7: Nuclear receptor coactivator 7
NCOR1: Nuclear receptor corepressor 1
NDD: Neurodevelopmental disorders
OXR1: Oxidation resistance 1
PIP: Phosphoinositide
SRC1: Steroid receptor coactivator 1
TLDc: Tre2/Bub2/Cdc16 (TBC), lysin motif (LysM), domain catalytic
TBC1D24: Tre2/Bub2/Cdc16 (TBC)1 domain family member 24
VGAT: Vesicular GABA transporter
VGLUT: Vesicular glutamate transporter
