# Supplementary material for: The Ncoa7 locus regulates V-ATPase formation and function, neurodevelopment and behaviour
Source: Cell Mol Life Sci. 2020 Dec 19;78(7):3503–24. doi: 10.1007/s00018-020-03721-6 (PMC8038996; doi:10.1007/s00018-020-03721-6)
Supplement: Supplementary file 1 — AUTHOR NOTE: The supplementary file 1 still contains the highlighted (in yellow) text from the resubmission that was added from the first submission. This needs to be converted to non-highlighted text before uploading pleaseSupplementary file1 (DOCX 22 KB) [file 18_2020_3721_MOESM1_ESM.docx]

**Supplementary Figure Legends and Table S1; Castroflorio E *et al.***

**Supplementary Figure S1:** *In situ* hybridisation showing developmental expression of *Ncoa7* in wild-type mice at embryonic day (E)15.5 and post-natal days (P)21 and P56. Expression is widespread in the developing brain including the regions indicated: Granule cell layer (gc) of the olfactory bulb (OB) and associated olfactory epithelium (OE), the cortical plate (CP) and the eventual cerebral cortical layers (CTX), diencephalon and eventual thalamus (TH), hippocampal formation (HPF), striatum (CP), piriform cortex (PF), entorhinal cortex (ENT) and hypothalamus (H). In the cerebellum, expression is high in the Purkinje cell layer (PCL; also see Supplementary Figure S2D), cerebellar nuclei (CBN) and brain stem medulla (M). The specificity of the probe is further demonstrated by very high levels of expression in the developing trigeminal ganglion (TGN). Scale bars: 400μm (E15.5), 1 mm (P21 and P56).

**Supplementary Figure S2. Generation, validation and neuropathological analysis of the *Ncoa7* DEL mutant**

**(A)** Schematic representation of the exon/intron structure of NCOA7, the NCOA7-B isoform and the targeted deletion in *Ncoa7-DEL* mice spanning across the entire gene (not to scale). **(B)** Representative PCR analysis of genomic DNA from WT, DEL and HET mice confirming the deletion of the *Ncoa7* locus. Relative positions of primers used are shown in (A). **(C)** Representative NCOA7 immunoblotting in WT and DEL mice from adult brain cerebral cortex tissue. **(D)** *In situ* hybridisation of *Ncoa7* mRNA in the adult brain of WT and DEL mice mice. Scale bar: 400 μm. **(E)** Quantification of total *Ncoa7* mRNA levels analysed by RT-PCR between the three genotypes from adult whole brain samples. **(F-K)** Morphometric analysis of the brain regions as indicated from WT and DEL mice at 12 months of age with representative confocal images of NeuN staining. (F-G) Motor (left), somatosensory (middle) and visual (right) cortices, scale bar: 200 μm; (H-J) CA1 pyramidal layer, CA3 piramidal layer and dentate gyrus granular layer in the hippocampus, scale bar: 100 μm; (J-K) Granule cell layer and molecular layer of the cerebellum, scale bar: 100 μm. **(L)** Quantification of Purkinje cell neuronal diameter and **(M)** density in the cerebellum of age-matched WT and DEL mice with representative confocal images of Purkinje neurons immunostained for calbindin **(N)**, scale bar: 200 μm. **(O)** Example caspase-3 immuostaining in the cerebellum from WT and DEL mice at 18 months of age with a positive control *Oxr1* knockout (*Oxr1^d/d^*) section at P22. Immunopositive cells are indicated (arrowheads) with the molecular layer (ML), Purkinke cell layer (PCL) and the granule cell layer (GCL) marked, Scale bar: 200 μm. All data are expressed as the mean +/- SEM. ****p* < 0.001, *****p* < 0.0001; one-way ANOVA / Bonferroni’s multiple comparison test, *n* = 4 WT and DEL mice (E and F-N).

**Supplementary Figure S3. *Ncoa7* deletion does not alter V-ATPase subunit expression *in vivo* and the protein is membrane-associated.**

Quantitative protein analysis by Western blot of **(A)** ATP6V0a1, **(B)** ATP6V1A1, **(C)** ATP6V1B2, **(D)** ATP6V1C1, **(E)** ATP6V1D1 subunits of the V-ATPase from whole cerebral cortex samples from adult age-matched WT and DEL mice. **(F)** Representative β3-tubulin western blot as loading control. Data are expressed as the mean +/- SEM. *n* = 5 for both WT and DEL samples.

**Supplementary Figure S4. *Ncoa7* deletion alters the number of LC3b-positive organelles in primary cortical neurons**

**(A)** Representative confocal images of cortical neurons (10-12 DIV) stained with anti-LC3b (green) and anti p62 (red) in WT (upper panel) and DEL (lower panel) mice. Scale bar: 10 μm **(B)** Quantitative analysis of LC3b fluorescence intensity and **(C)** number of LC3b-positive organelles. **(D)** Quantitative analysis of p62 fluorescence intensity and **(E)** number of p62-positive organelles. Data are expressed as the mean +/- SEM and data points represent cells from 3 independent preparations. **p* < 0.05; unpaired Student’s t-test.

**Supplementary Figure S5. *Ncoa7* deletion influences the expression of lysosomal markers in the brain**

(**A**) Representative immunoblotting of endosomal and lysosomal markers. WB analysis of **(B)** LAMP1, **(C)** RAB7, **(D)** CathB, **(E)** Pro-CathD and **(F)** CathD heavy chain for age-matched WT and DEL whole cerebral cortex tissue with β3-tubulin as the loading control. Data are expressed as the mean +/- SEM and data points represent individual animals. **p* < 0.05; unpaired Student’s t-test.

**Supplementary Figure S6. Influence of *Ncoa7* deletion in the kidney**

**(A)** Western blotting for NCOA7 (top panel) and ATP6V1B1 (bottom panel) after immunopreciptation (IP) from whole adult kidney extracts from WT and DEL mice using the reciprocal antibodies as indicated. Blots of equally loaded protein extracts prior to IP (input) are also shown. **(B-C)** Quantification of ATP6V1B1 levels from whole adult kidney extracts against total protein loading. **(D)** Urine pH measurements from adult WT and DEL mice, *n* = 26-27 per genotype. Data are expressed as the mean +/- SEM and data points represent individual animals. **p* < 0.05; unpaired Student’s t-test.

**Supplementary Figure S7. Behavioural test battery of *Ncoa7* deletion mice**

**(A-B)** Spontaneous activity in the open field as (A) total distance travelled and (B) mean velocity in a 20 min trial. **(C)** Rotarod testing over three consecutive days as latency before falling. **(D-G)** Spatial novelty preference in the Y-maze, showing: (D) familiar arm duriation and (E) frequency of visits during the habituation (green) and novelty (orange) aspects of the task and (F) novel arm duration and (G) frequency of visits during the novelty aspect of the test. **(H-M)** Fear conditioning testing, showing: (H) Number of freezing episodes and (I) freezing time during the fear conditioning training, (J) number of freezing episodes and (K) freezing time during the cue fear conditioning test, and (L) number of freezing episodes and (M) freezing time in the context fear conditioning aspect of the test. **(N)** Three-chamber social testing data showing total distance moved in the habituation (green) and novelty (orange) aspects of the test. **(O)** Social dominance tube test as percentage of ‘wins’ over all trials. Data are expressed as the mean +/- SEM. ****p* < 0.001; one-way ANOVA / Bonferroni’s multiple comparison test.

**Supplementary Figure S8.** **The effect of heterozygosity of *Ncoa7* in addition to loss of *Oxr1* on the pathology in the cerebellum**

**(A)** Caspase-3 immunostaining from cerebellar sections of WT (*Oxr1^+/+^ / Ncoa7^+/+^*) and the two Oxr1 knockout (*Oxr1^d/d^*) mutant lines as indicated. Scale bar: 100 m. The data are quantified in **(B)**, *n* = 4 animals per genotype. Data are expressed as the mean +/- SEM; unpaired Student’s t-test.

**Supplementary Table 1.** Offspring from intercross of *Oxr1* knockout and *Ncoa7* deletion lines with binomial probability of each genotype number obtained versus expected Mendelian ratios

|  | **expected** | **identified** | **probability** |
| --- | --- | --- | --- |
| P22 genotypes from *Oxr1^d/+^ / Ncoa7^del/+^ x Oxr1^d/+^ / Ncoa7^del/+^* | | | |
| *Oxr1^+/+^ / Ncoa7^+/+^* | 8.5 | 17 | 0.0029 |
| *Oxr1^+/+^ / Ncoa7^del/+^* | 17 | 23 | 0.0301 |
| *Oxr1^+/+^ / Ncoa7^del/del^* | 8.5 | 8 | 0.1416 |
| *Oxr1^d/+^ / Ncoa7^+/+^* | 17 | 25 | 0.0129 |
| *Oxr1^d/+^ / Ncoa7^del/+^* | 34 | 26 | 0.0229 |
| *Oxr1^d/+^ / Ncoa7^del/del^* | 17 | 19 | 0.0862 |
| *Oxr1^d/d^ / Ncoa7^+/+^* | 8.5 | 8 | 0.1416 |
| *Oxr1^d/d^ / Ncoa7^del/+^* | 17 | 10 | 0.0195 |
| *Oxr1^d/d^ / Ncoa7^del/del^* | 8.5 | 0 | 0.0002 |
| **Total** | **136** | **136** |  |
|  |  |  |  |
| E8.5-9.5 genotypes from *Oxr1^d/+^ / Ncoa7^del+^* x *Oxr1^d/+^ / Ncoa7^del/del^* | | | |
| *Oxr1^+/+^ / Ncoa7^del/+^* | ~10 | 11 | 0.1231 |
| *Oxr1^+/+^ / Ncoa7^del/del^* | ~10 | 14 | 0.0540 |
| *Oxr1^d/+^ / Ncoa7^del/+^* | ~20 | 18 | 0.0894 |
| *Oxr1^d/+^ / Ncoa7^del/del^* | ~20 | 26 | 0.0338 |
| *Oxr1^d/d^ / Ncoa7^del/+^* | ~10 | 12 | 0.1026 |
| *Oxr1^d/d^ / Ncoa7^del/del^* | ~10 | 0 | <0.0001 |
| **Total** | **81** | **81** |  |
