# Supplementary figures and images for: The Ncoa7 locus regulates V-ATPase formation and function, neurodevelopment and behaviour
Source: Cell Mol Life Sci. 2020 Dec 19;78(7):3503–24. doi: 10.1007/s00018-020-03721-6 (PMC8038996; doi:10.1007/s00018-020-03721-6)

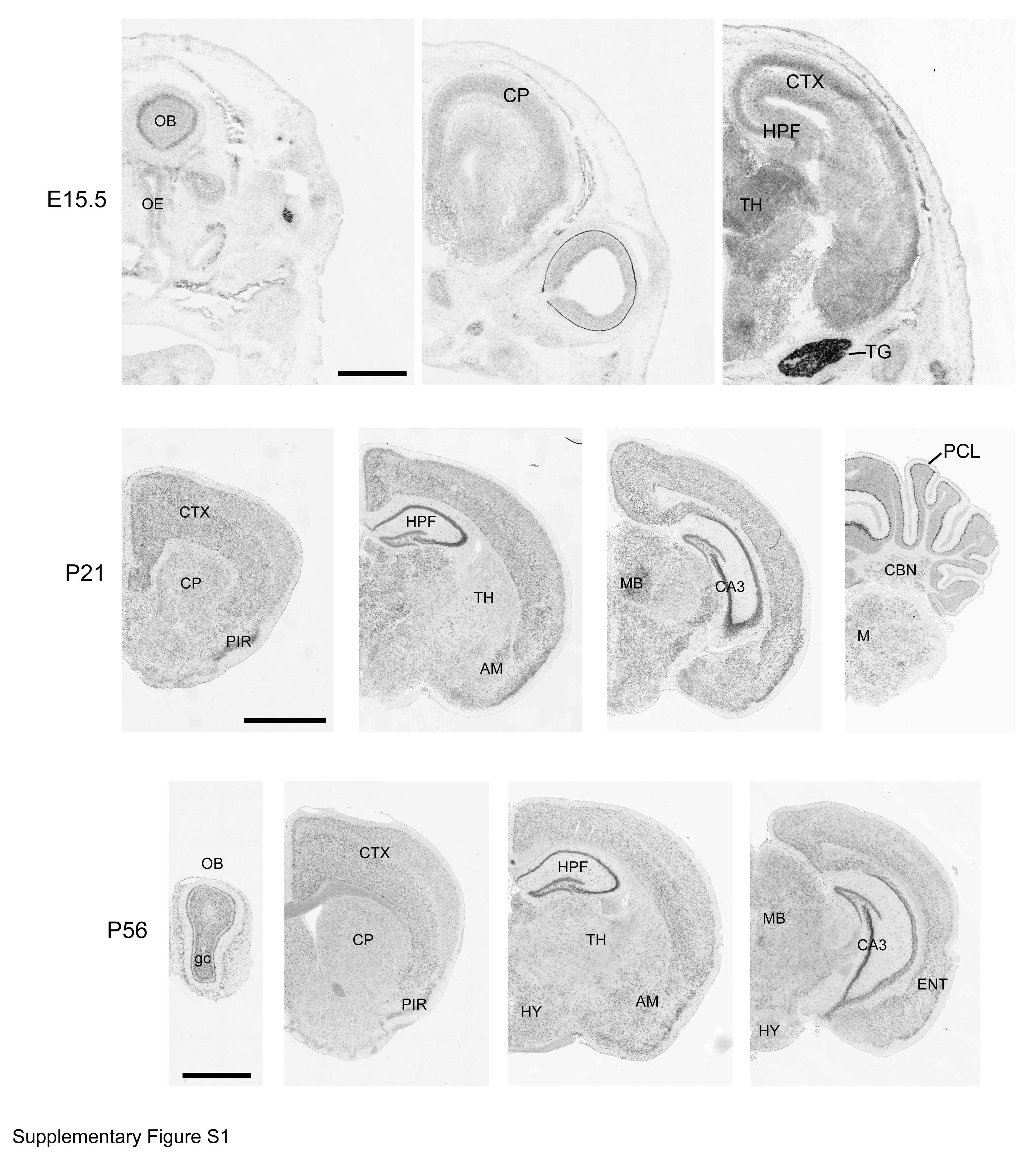

Supplement: Supplementary file 2 — Supplementary file2 (TIF 5259 KB) [file 18_2020_3721_MOESM2_ESM.tif]

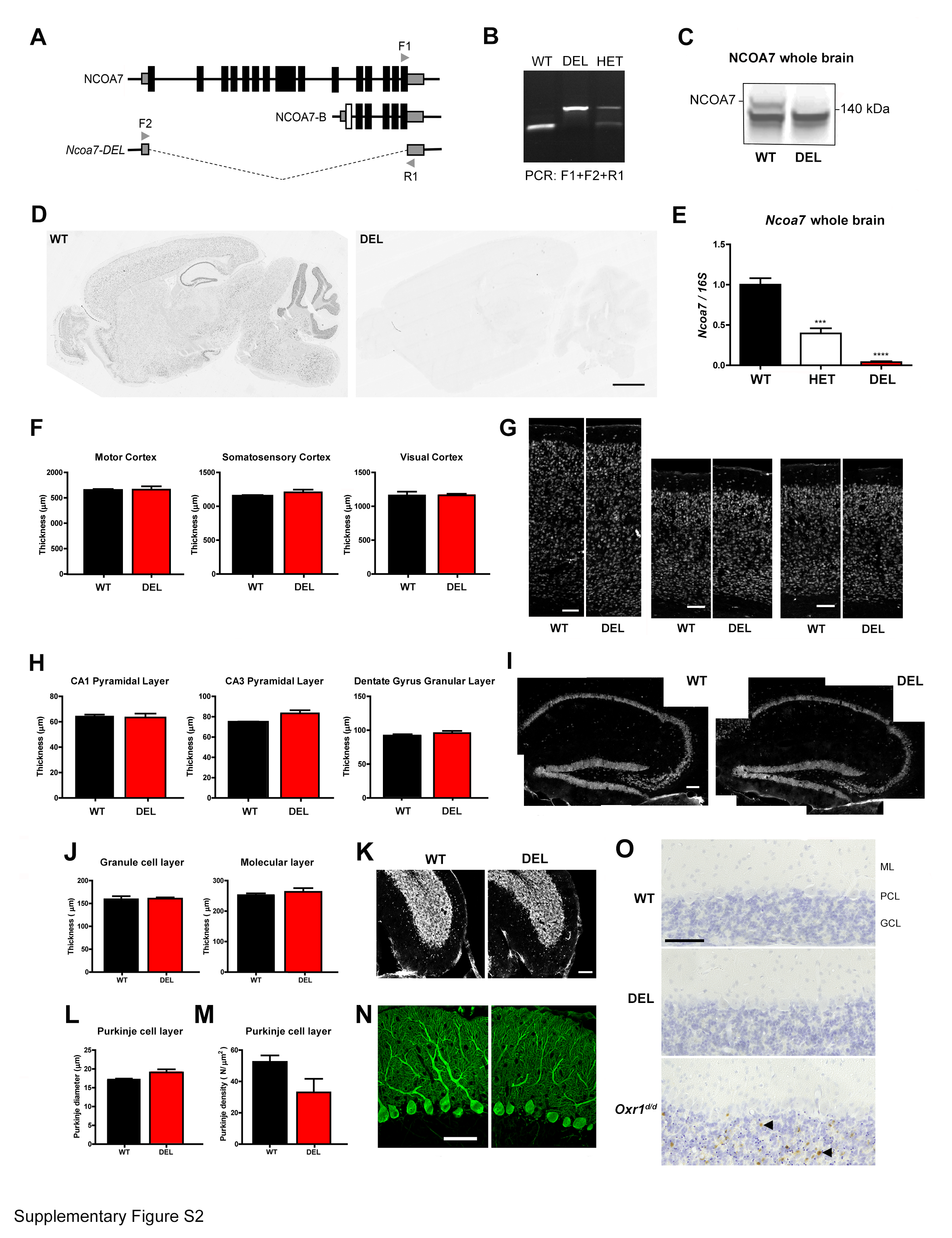

Supplement: Supplementary file 3 — Supplementary file3 (TIF 3119 KB) [file 18_2020_3721_MOESM3_ESM.tif]

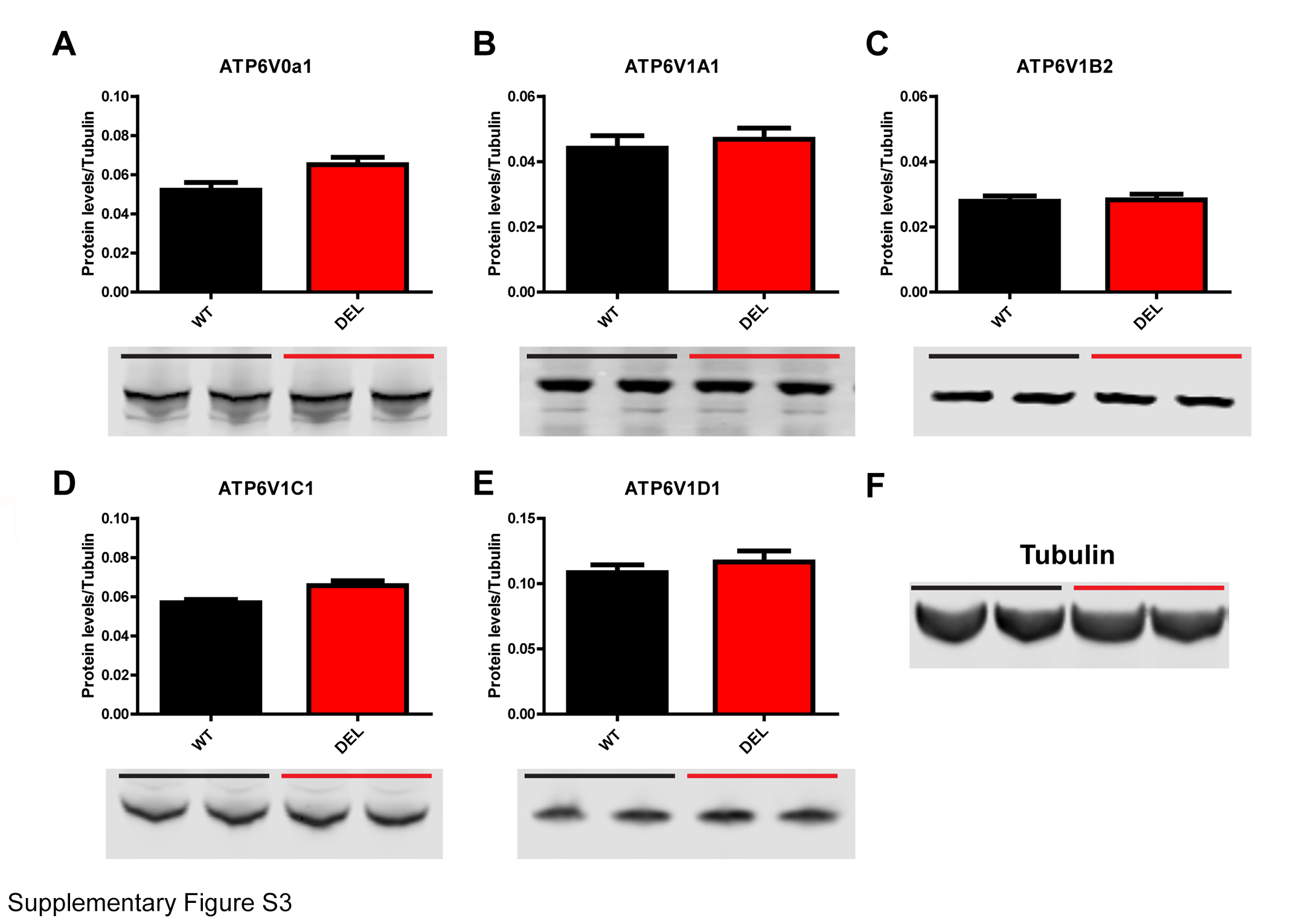

Supplement: Supplementary file 4 — Supplementary file4 (TIF 360 KB) [file 18_2020_3721_MOESM4_ESM.tif]

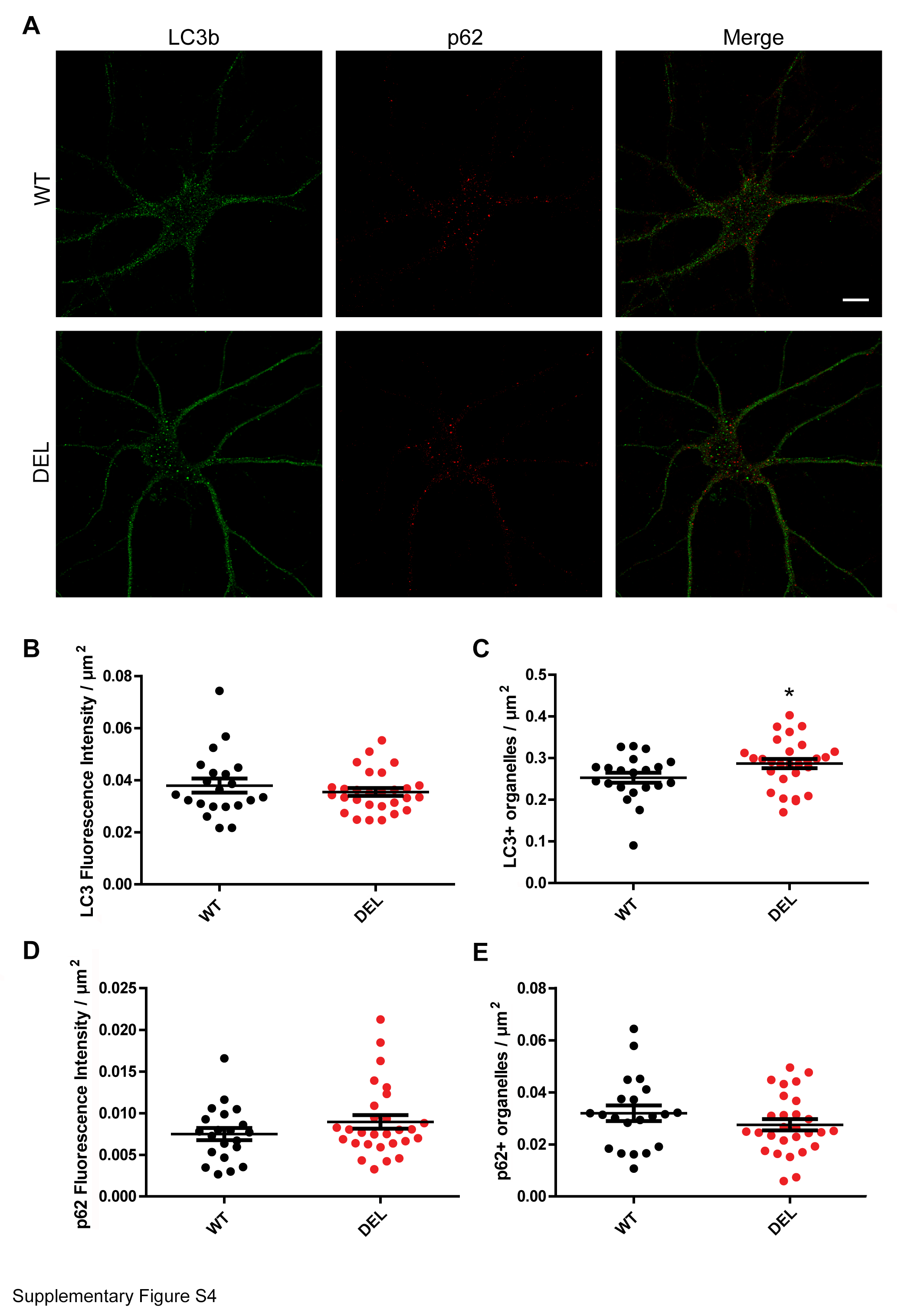

Supplement: Supplementary file 5 — Supplementary file5 (TIF 802 KB) [file 18_2020_3721_MOESM5_ESM.tif]

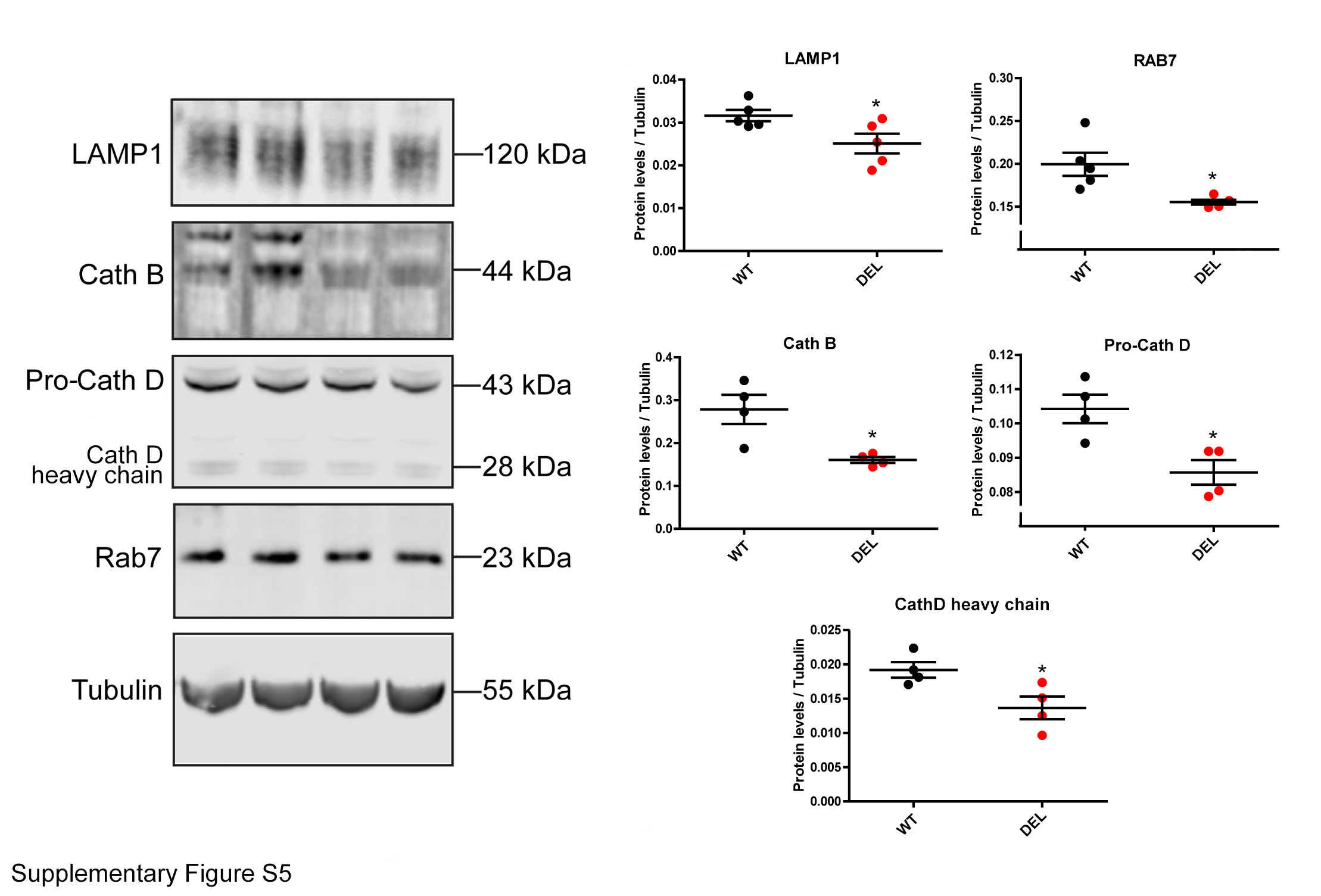

Supplement: Supplementary file 6 — Supplementary file6 (TIF 407 KB) [file 18_2020_3721_MOESM6_ESM.tif]

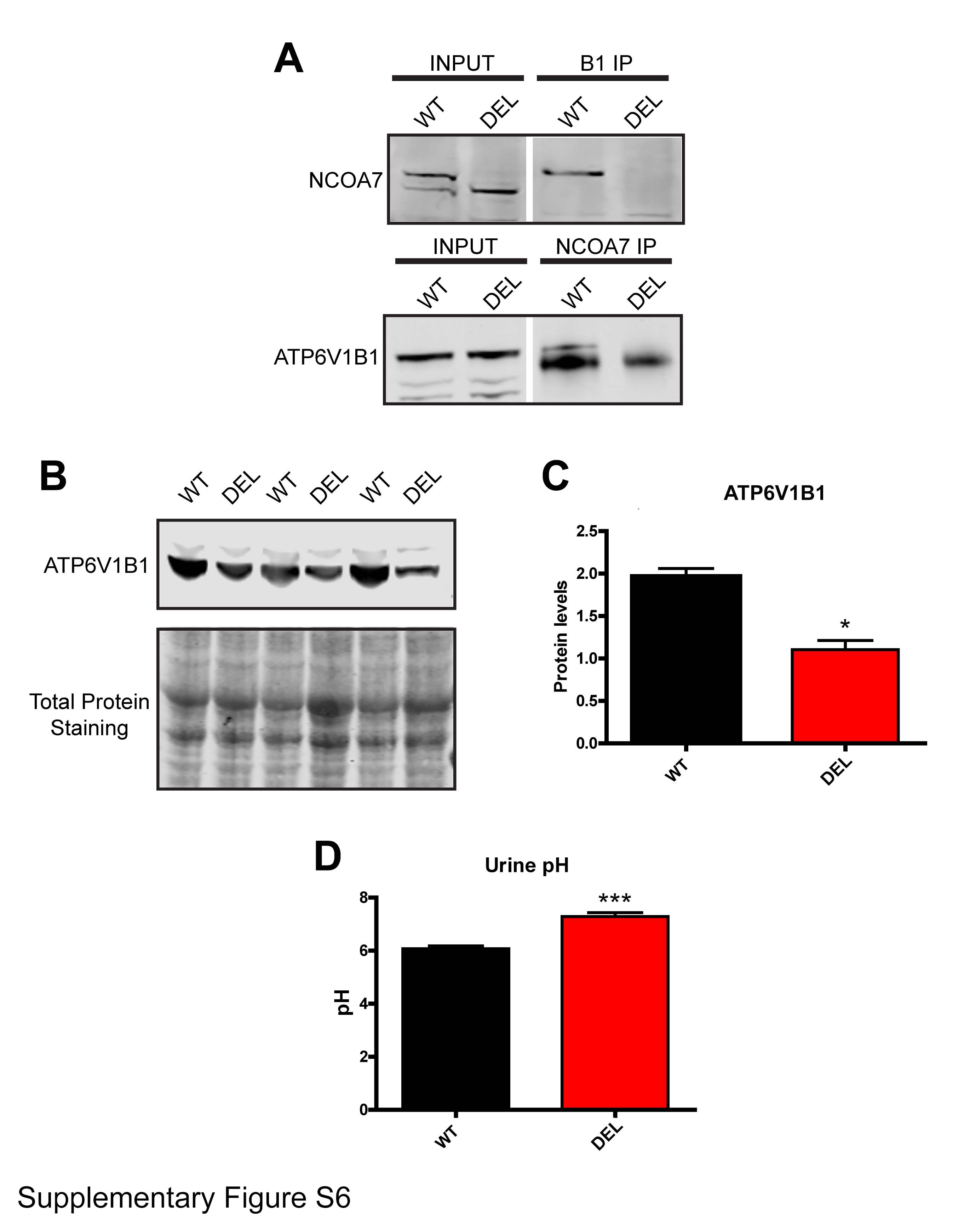

Supplement: Supplementary file 7 — Supplementary file7 (TIF 618 KB) [file 18_2020_3721_MOESM7_ESM.tif]

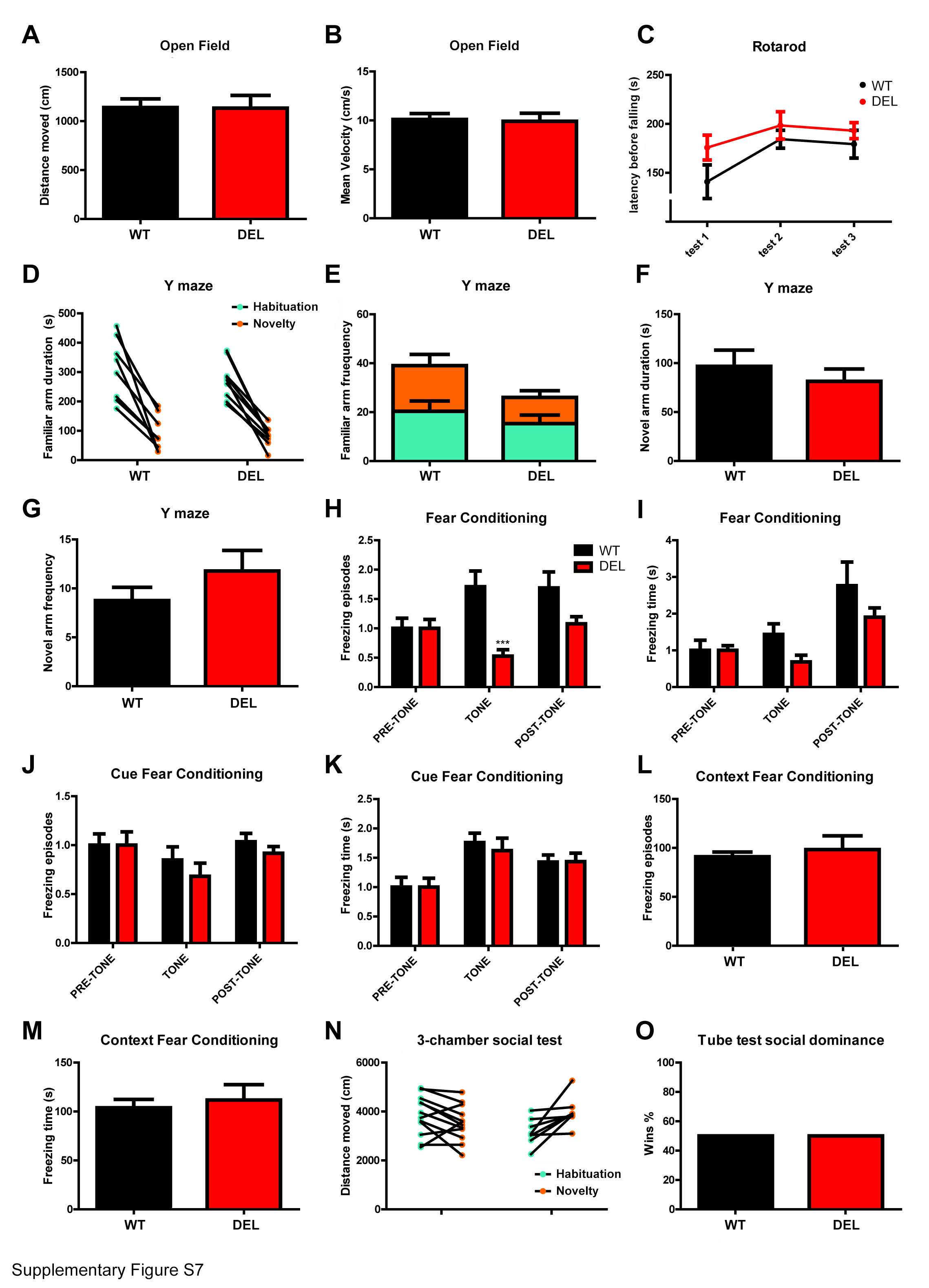

Supplement: Supplementary file 8 — Supplementary file8 (TIF 409 KB) [file 18_2020_3721_MOESM8_ESM.tif]

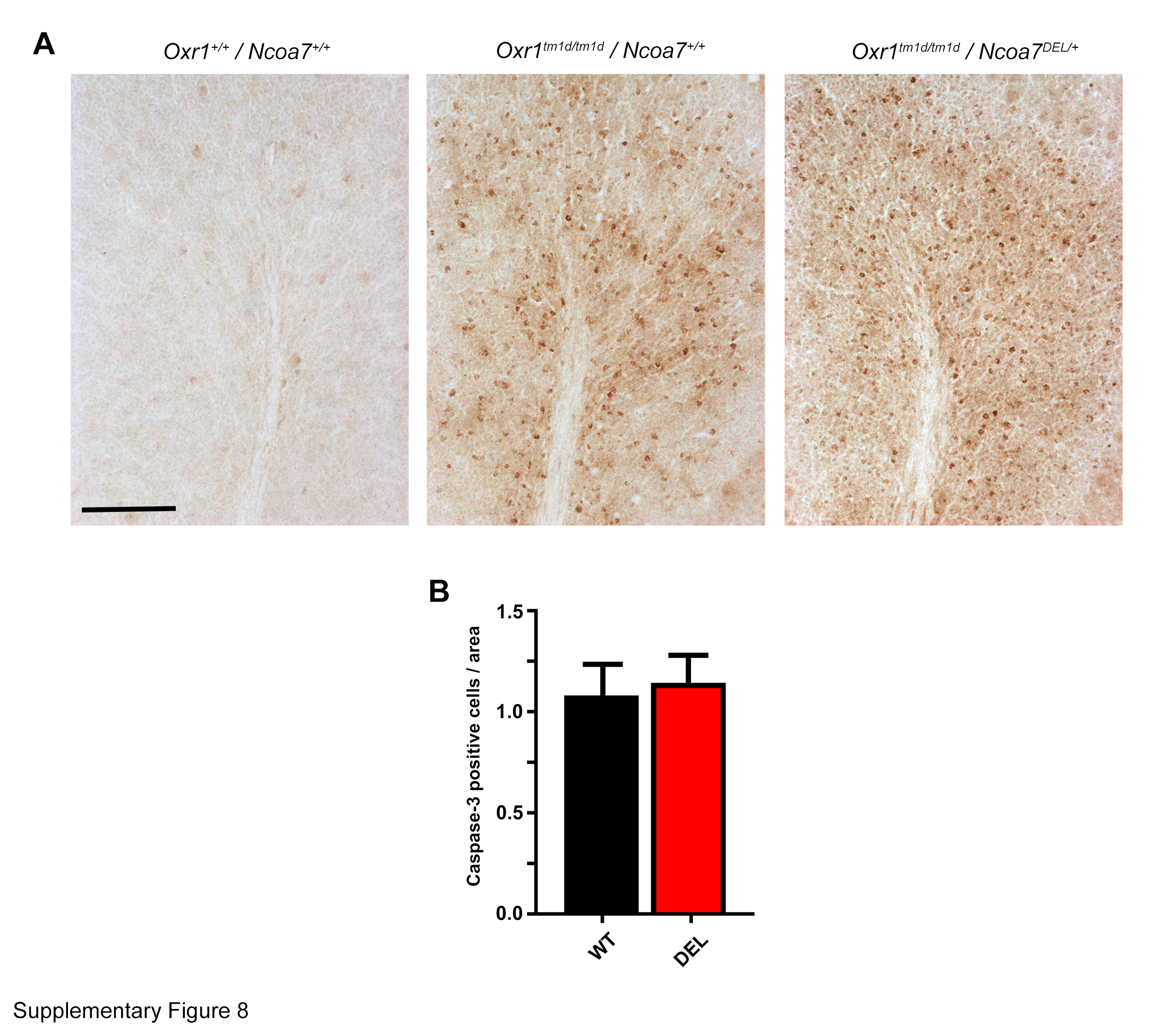

Supplement: Supplementary file 9 — Supplementary file9 (TIF 9688 KB) [file 18_2020_3721_MOESM9_ESM.tif]
